# Foldable anode-free sodium batteries enabled by N,P-codoped carbon macroporous fibers incorporated with CoP nanoparticles

**DOI:** 10.1126/sciadv.adv2007

**Published:** 2025-05-09

**Authors:** Yongling An, Zhihao Pei, Deyan Luan, Xiong Wen (David) Lou

**Affiliations:** Department of Chemistry, City University of Hong Kong, 83 Tat Chee Avenue, Kowloon 999077, Hong Kong, China.

## Abstract

Anode-free sodium batteries (AFNBs) are regarded as promising alternatives for next-generation energy storage systems because of their high energy density, high safety, cost-effectiveness, and simple manufacturing processes. However, their practical application is limited by a low coulombic efficiency (CE) and a short lifespan resulting from a large volume change and the growth of Na dendrites. Here, we propose a three-dimensional versatile host composed of interconnected N,P-codoped carbon macroporous fibers incorporated with CoP nanoparticles (denoted as CoP@N/P-CMFs) for selective nucleation and uniform deposition of Na. The porous skeleton reduces structural stress and inhibits dendrite growth by decreasing local current density and homogenizing Na^+^ flux. Furthermore, the sodiophilic CoP and N,P-codoped carbon reduce the energy barrier for Na nucleation, further manipulating homogeneous Na growth. Consequently, the CoP@N/P-CMFs exhibit an ultrahigh CE of more than 99.97% (10 milliamperes per square centimeter and 10 milliampere-hours per square centimeter) and dendrite-free Na deposition. When coupled with a Na_3_V_2_(PO_4_)_3_ cathode, the assembled foldable AFNBs demonstrate stable cycling performance.

## Teaser

N,P-codoped carbon macroporous fibers with CoP nanoparticles serve as a flexible host for foldable anode-free Na batteries.

## INTRODUCTION

Sodium-ion batteries are considered as economical technologies for energy storage owing to the natural abundance of Na resources (*1*–*3*). However, their energy density is relatively low as Na ions, with large atomic weight, serve as the charge carrier (*4*–*6*). Designing bare current collectors to construct anode-free devices can maximize energy density, showing great promise for accelerating commercialization applications (*7*–*9*). In addition to high energy density, anode-free Na batteries (AFNBs) offer several advantages, including high safety, low cost, and simple manufacturing processes (*10*–*12*). However, achieving high energy density also places stringent demands on the coulombic efficiency (CE) of Na metal on the current collector, as no additional Na is incorporated into the battery (*13*–*15*). In AFNBs, the CE is primarily compromised by factors such as an unstable solid electrolyte interphase (SEI) layer, huge volume change, and rampant dendrite growth (*16*–*18*).

To circumvent these challenges, many strategies have been developed to improve CE by designing three-dimensional (3D) current collectors (*19*–*23*), modifying artificial protective layers (*14*, *24*–*27*), exploiting alternative current collectors (*28*, *29*), optimizing functional electrolytes (*9*, *17*, *30*), and so on. On the basis of classical diffusion principles, decreasing the local current density can mitigate charge accumulation and concentration polarization, thereby inhibiting the Na dendrite growth induced by the tip effect and further improving CE (*10*). In this context, building 3D conductive current collectors is viewed as a viable method to manipulate Na^+^ flux, further hindering dendrite formation and controlling the Na plating/stripping process (*11*). Currently, many 3D scaffolds have been designed as reliable current collectors for AFNBs, such as porous Al (*28*), porous carbon (*20*, *21*), etc. To further reduce the interfacial energy between current collectors and deposited Na metals, sodiophilic species with a strong Na affinity have been integrated to regulate Na nucleus growth and achieve uniform Na deposition (*10*, *31*). For instance, doping carbon materials with heteroatoms (e.g., nitrogen, oxygen, and sulfur) can decrease the energy barriers for Na nucleation and uniformize Na^+^ flux (*21*, *32*). Moreover, Na-reactive materials, such as Au (*33*), Ag (*34*), Sn (*35*), Sb (*36*), Co_3_O_4_ (*37*), and SnO_2_ (*38*), can improve sodiophilicity to promote homogeneous Na nucleation. Consequently, designing a multifunctional current collector with multifarious advantages (abundant high-affinity Na binding sites and 3D macroporous framework) may realize stable AFNBs with high energy density and long lifespan.

In this work, N,P-codoped carbon macroporous fibers incorporated with CoP nanoparticles (denoted as CoP@N/P-CMFs) are developed as a current collector for high-energy AFNBs. Benefitting from the 3D conductive framework, the CoP@N/P-CMFs not only homogenize Na deposition by decreasing local current density and regulating Na^+^ flux distribution but also provide enough space to accommodate volume change and confine Na plating. In addition, the CoP nanoparticles and N,P-codoped carbon, which have a high affinity for Na^+^ ions and a low nucleation barrier, can manipulate uniform Na nucleation and growth. These compositional and structural benefits endow the CoP@N/P-CMFs scaffold with low polarization and high CE (99.97%) at 10 mA cm^−2^ and 10 mAh cm^−2^. Furthermore, a foldable anode-free pouch cell with a Na_3_V_2_(PO_4_)_3_ (NVP) cathode and CoP@N/P-CMFs host is assembled, demonstrating improved cycling performance and good rate capability.

## RESULTS

### Fabrication and characterization of CoP@N/P-CMFs

**Fig. 1.**
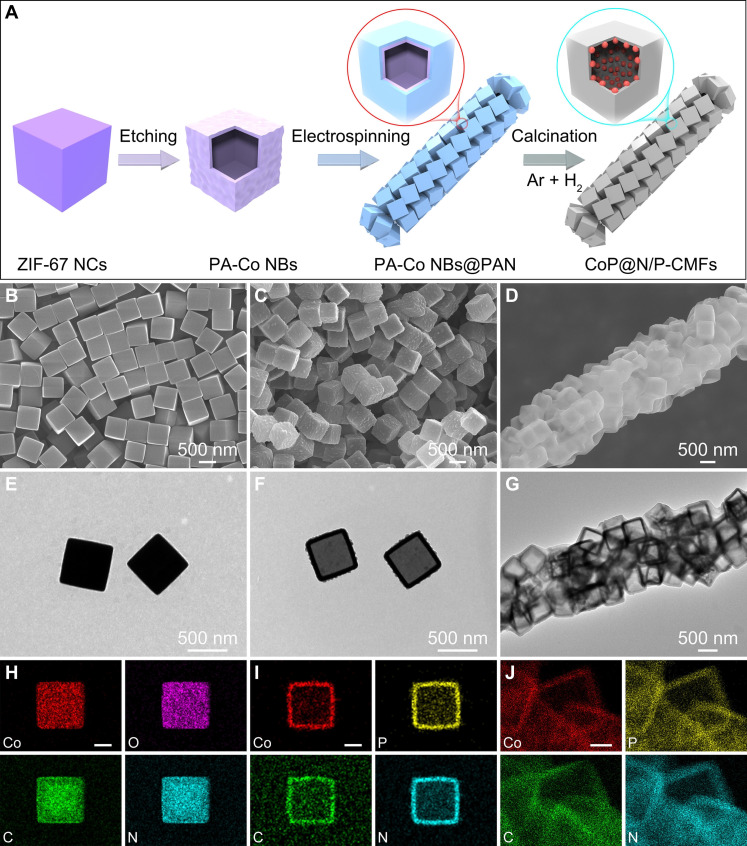
Formation process of the CoP@N/P-CMFs. (**A**) Schematic representation of the fabrication process for CoP@N/P-CMFs. (**B** to **D**) FESEM, (**E** to **G**) TEM, and (**H** to **J**) elemental mapping images of [(B), (E), and (H)] ZIF-67 NCs, [(C), (F), and (I)] PA-Co NBs, and [(D), (G), and (J)] PA-Co@PAN. Scale bars, 200 nm [(H) to (J)].

CoP@N/P-CMFs are fabricated using a template-engaged technique (Fig. 1A). Initially, homogeneous zeolitic imidazolate framework-67 nanocubes (ZIF-67 NCs) are used as the starting material. The size distribution of the ZIF-67 NCs can be controlled by varying the quantity of cetyltrimethylammonium bromide (CTAB) introduced (figs. S1 and S2). ZIF-67 NCs, averaging around 610 nm in size, function as the precursor (Fig. 1, B, E, and H). Then, ZIF-67 NCs are etched using a phytic acid (PA) solution to synthesize PA-Co nanoboxes (NBs) (*39*). The PA-Co NBs exhibit a cubic structure with a hollow interior (Fig. 1, C, F, I, and fig. S3). Subsequently, the PA-Co NBs are electrospun with polyacrylonitrile (PAN) to obtain PA-Co@PAN fibers. Compared to that of PAN (figs. S4 and S5), scanning electron microscopy (SEM) and transmission electron microscopy (TEM) images of PA-Co@PAN display that the PA-Co NBs are densely enclosed inside the PAN fibers along the long axis (Fig. 1, D, G, and J, and figs. S6 and S7).

**Fig. 2.**
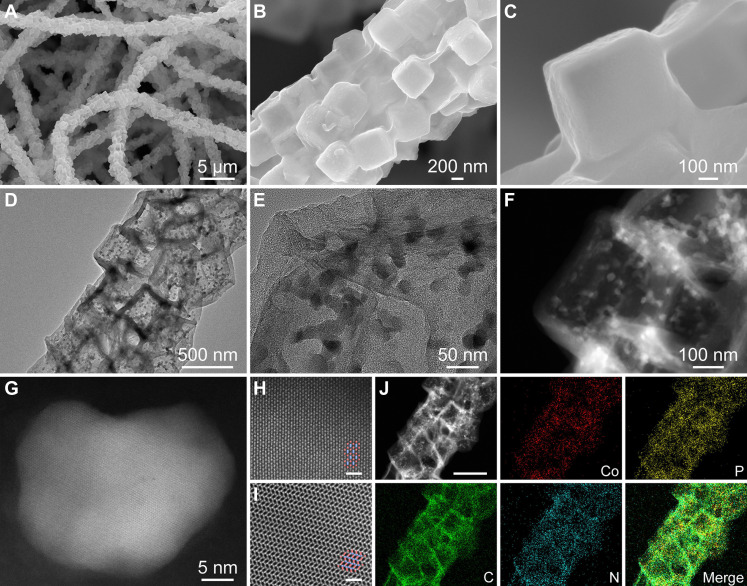
Morphological and structural characterizations. (**A** to **C**) FESEM, (**D** and **E**) TEM, (**F** to **I**) HAADF-STEM, and (**J**) elemental mapping images of CoP@N/P-CMFs. Scale bars, 1 nm [(H) and (I)] and 500 nm (J).

After the carbonization process, the obtained CoP@N/P-CMFs inherit a thickness of approximately 91 μm and a 1D fibrous morphology with a diameter of about 1.8 μm (Fig. 2, A to C, and fig. S8). The TEM image exhibits a hierarchical hollow structure with cubic voids and substantial pores (Fig. 2D). In particular, some irregular nanoparticles are attached on the internal shells (Fig. 2E). The high-angle annular dark-field scanning TEM (HAADF-STEM) technique is used to further explore the structure of CoP@N/P-CMFs. Figure 2F clearly displays the CoP nanoparticles pinned into the carbon substrate, which coincides with the result of the field-emission SEM (FESEM) images. Atomic-resolution HAADF images of CoP@N/P-CMFs show clear lattice fringes of CoP, verifying its high crystallinity (Fig. 2, G to I). Elemental mapping images illustrate a uniform distribution of C, N, P, and Co elements in the CoP@N/P-CMFs (Fig. 2J). The x-ray diffraction (XRD) pattern (fig. S9) of CoP@N/P-CMFs can be indexed as CoP (JCPDS card no. 29-0497), and the energy-dispersive x-ray (EDX) spectrum validates the presence of C, N, Co, and P elements (*39*, *40*). The Raman spectra exhibit two distinctive peaks ascribed to the G band and D band of carbon, corresponding to sp2 carbon and disordered carbon, respectively (fig. S10) (*41*). The carbon content is calculated to be about 34.6% (fig. S11). The Brunauer-Emmett-Teller (BET) surface area of CoP@N/P-CMFs is around 132.1 m^2^ g^−1^ (fig. S12). To explore the surface valence state of CoP@N/P-CMFs, an x-ray photoelectron spectroscopy (XPS) test is executed (fig. S13). In the high-resolution Co 2p XPS spectrum, two peaks located at 778.8 and 794.0 eV are assigned to Co-P 2p_3/2_ and Co-P 2p_1/2_, respectively (*42*). Besides, the peaks located at 782.4 and 798.3 eV combined with the shakeup satellite peaks (denoted as Sat. at 786.5 and 803.5 eV) imply the existence of Co-O with a Co^2+^ oxidation state (*43*). The deconvolution of the high-resolution P 2p spectrum displays three peaks located at 129.9, 132.6, and 133.9 eV, corresponding to Co-P, P-C, and P-O, respectively (*41*). In the high-resolution N 1 s spectrum, four peaks at 398.6, 400.4, 401.6, and 404.7 eV correspond to pyridinic N, pyrrolic N, quaternary N, and oxidized N, respectively (*44*). Optical photographs of PA-Co@PAN and CoP@N/P-CMFs evidence the excellent flexibility of CoP@N/P-CMFs (fig. S14).

**Fig. 3.**
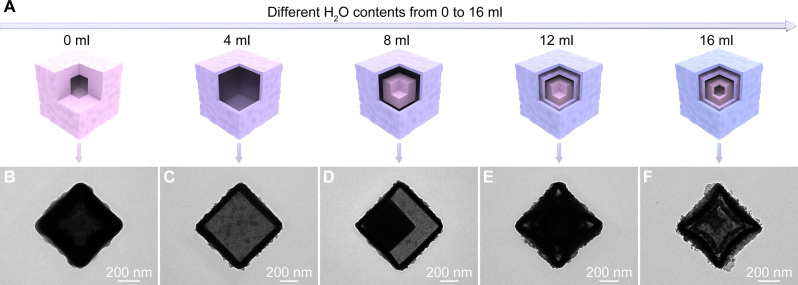
Morphological evolution of PA-Co. (**A**) Schematic representation of the formation process of the PA-Co hollow structure with multiple shells. (**B** to **F**) TEM images of PA-Co obtained after etching with different DIW contents in an ethanol/DIW solution: (B) 0 ml, (C) 4 ml, (D) 8 ml, (E) 12 ml, and (F) 16 ml.

To acquire further insights into the evolution of PA-Co, the products etched from the ZIF-67 precursor with different deionized water (DIW) contents in an ethanol/DIW solution are characterized (Fig. 3A and fig. S15). At a DIW content of 0 ml, a single-shelled hollow structure with a thick shell is synthesized (Fig. 3B). When the DIW content increases to 4 ml, the obtained PA-Co displays a single-shelled hollow structure with a thin shell (Fig. 3C). Further increasing the DIW content to 8 ml leads to the formation of a double-shelled hollow structure with a solid core (Fig. 3D). Increasing the DIW content to 12 ml results in a triple-shelled hollow structure with a solid core (Fig. 3E). When the DIW content reaches 16 ml, the original solid NCs evolve into a triple-shelled hollow structure with a porous core (Fig. 3F). Thus, the hollow structure of PA-Co with multiple shells can be regulated by adjusting the DIW content in the etching solution.

### Theoretical calculation and Na deposition investigation

**Fig. 4.**
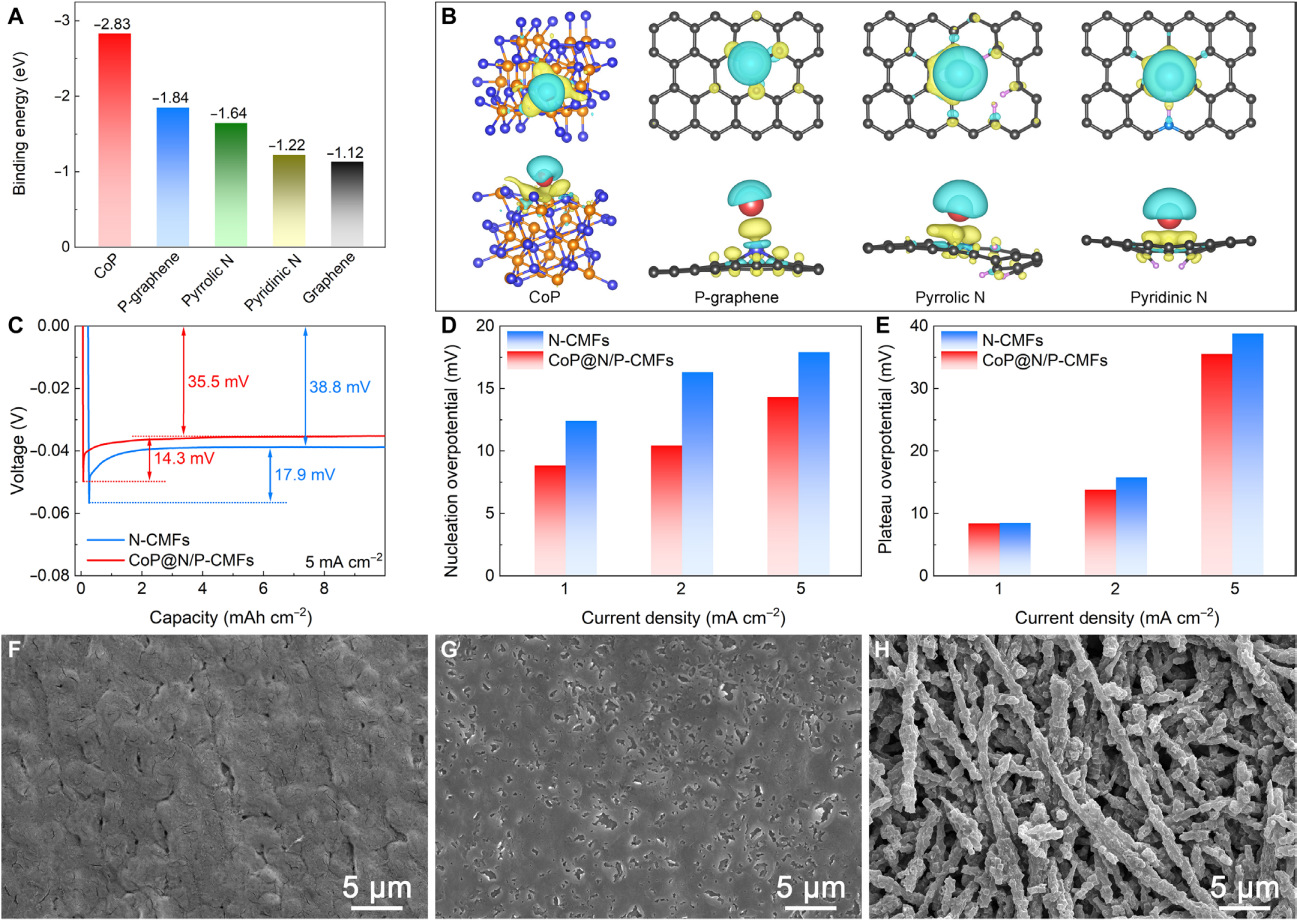
Theoretical calculations and Na deposition investigations. (**A**) Summary of the calculated binding energy of Na atoms with CoP, graphene, P-doped graphene, and N-doped graphene. (**B**) Interfacial charge-density models of CoP, P-doped graphene, and N-doped graphene with Na atom adsorption (cyan: depletion; yellow: accumulation). (**C**) Galvanostatic discharge profiles during Na nucleation on various hosts tested at 5 mA cm^−2^. (**D**) Nucleation overpotential and (**E**) plateau overpotential of various hosts tested at 1, 2, and 5 mA cm^−2^. (**F** and **G**) FESEM images of CoP@N/P-CMFs after Na deposition at different capacities of (F) 10 mAh cm^−2^ and (G) 20 mAh cm^−2^. (**H**) FESEM image of CoP@N/P-CMFs after Na stripping at a capacity of 20 mAh cm^−2^.

Density functional theory calculations are executed to probe the significance of each constituent in CoP@N/P-CMFs (figs. S16 and S17). Sodiophilicity involves the capability to absorb and bond with Na, thereby reducing the energy barrier for Na nucleation (*19*, *45*, *46*). The calculated binding energies between Na atoms and different species within CoP@N/P-CMFs are summarized in Fig. 4A. The CoP species exhibit a higher binding energy compared to other species, indicating a robust Na affinity. Moreover, all P- and/or N-doped graphene products display increased binding energy compared to pristine graphene, verifying the improved sodiophilicity of heteroatom-doped carbon. In addition, the differential charge density models reveal a robust interaction between Na atoms and CoP, P-graphene, pyrrolic N, and pyridinic N, validating visible charge transfer at the interface (Fig. 4B).

The initial nucleation process is crucial for determining the quality of Na plating (*20*, *47*). Therefore, the nucleation overpotential of Na on various hosts is investigated under different current densities. In contrast, N-doped carbon macroporous fibers (referred to as N-CMFs) are prepared using a similar process (figs. S18 and S19). Compared to other hosts, CoP@N/P-CMFs display a low Na nucleation overpotential at different current densities (Fig. 4, C to E, and fig. S20). Besides, the plateau overpotential of CoP@N/P-CMFs is lower than that of the other hosts, indicating improved Na ion migration to the electrode surface. Specifically, the nucleation overpotential and plateau overpotential of CoP@N/P-CMFs are 14.3 and 35.5 mV at 5 mA cm^−2^, respectively, which are smaller than that of N-CMFs. The homogeneously distributed CoP nanoparticles with improved sodiophilicity ensure an expeditious and uniform Na nucleation behavior in the macroporous 3D matrix, substantially decreasing the nucleation barrier and accelerating the reaction dynamics. Besides, the functional N,P-codoped carbon sites also improve the sodiophilic nature to guide uniform heterogeneous Na nucleation and growth behavior (*48*).

The morphological and structural evolution of CoP@N/P-CMFs after Na deposition and dissolution is probed. The charge/discharge voltage curves of the CoP@N/P-CMFs host at 1 mA cm^−2^ are presented (fig. S21). No Na dendrites are observed when the Na plating capacity reaches 1 mAh cm^−2^. With further deposition, apparent Na lumps instead of dendritic structures are detected. When the Na deposition capacity increases to 4 mAh cm^−2^, the deposited Na is observed on both the exterior and inner surfaces of the hollow fibers without any visible Na dendrites. Benefiting from the abundant sodiophilic sites and small overpotential for nucleation and growth, the CoP@N/P-CMFs enable homogeneous Na growth on both the exterior and interior surfaces of the hollow fibers. Besides, the 3D porous scaffold offers abundant space to deposit Na metal within the host and inhibit volume change during cycling. During the following charging process, Na is reversibly stripped from the CoP@N/P-CMFs host, accompanied by the gradual vanishment of Na metal and the recovery of the nanofiber structure. After charging to 1 V, Na is nearly completely stripped from the CoP@N/P-CMFs host without any “dead Na” residue, while the 3D macroporous morphology is well maintained. Besides, the morphological changes of CoP@N/P-CMFs at high plating capacities are also investigated. At a Na plating capacity of 10 mAh cm^−2^, the 3D macroporous network of the CoP@N/P-CMFs host gradually fills to form a smooth surface (Fig. 4F and fig. S22). At a higher Na deposition capacity of 20 mAh cm^−2^, the CoP@N/P-CMFs display a dense and smooth surface, indicating that CoP@N/P-CMFs homogenize Na deposition (Fig. 4G and fig. S23). After Na stripping to 1 V, Na metal is nearly entirely stripped from the CoP@N/P-CMFs and the framework is restored to its 3D structure (Fig. 4H). In contrast, the surface of N-CMFs host is covered with irregularly aligned flakes after Na plating to 10 mAh cm^−2^ (fig. S24). As the Na deposition capacity improves to 20 mAh cm^−2^, the N-CMFs show a coarse surface with dendritic structures (fig. S25). After charging to 1 V, most Na metal is stripped, but some remains on the N-CMFs host. The superior regulation of Na plating and stripping in the CoP@N/P-CMFs host can be ascribed to its 3D macroporous structure and abundant sodiophilic sites. Specifically, the 3D macroporous framework homogenizes ion flux distributions, reduces local current density, and alleviates volume variation, thus achieving uniform Na deposition and high plating capacity (*19*, *20*). The functional sodiophilic CoP nanoparticles and N,P-codoped carbon lower the nucleation barrier and increase interaction with Na^+^, thereby regulating the homogeneous Na growth on both the exterior and interior surfaces of the hollow fibers (*24*, *36*).

### Electrochemical performance

**Fig. 5.**
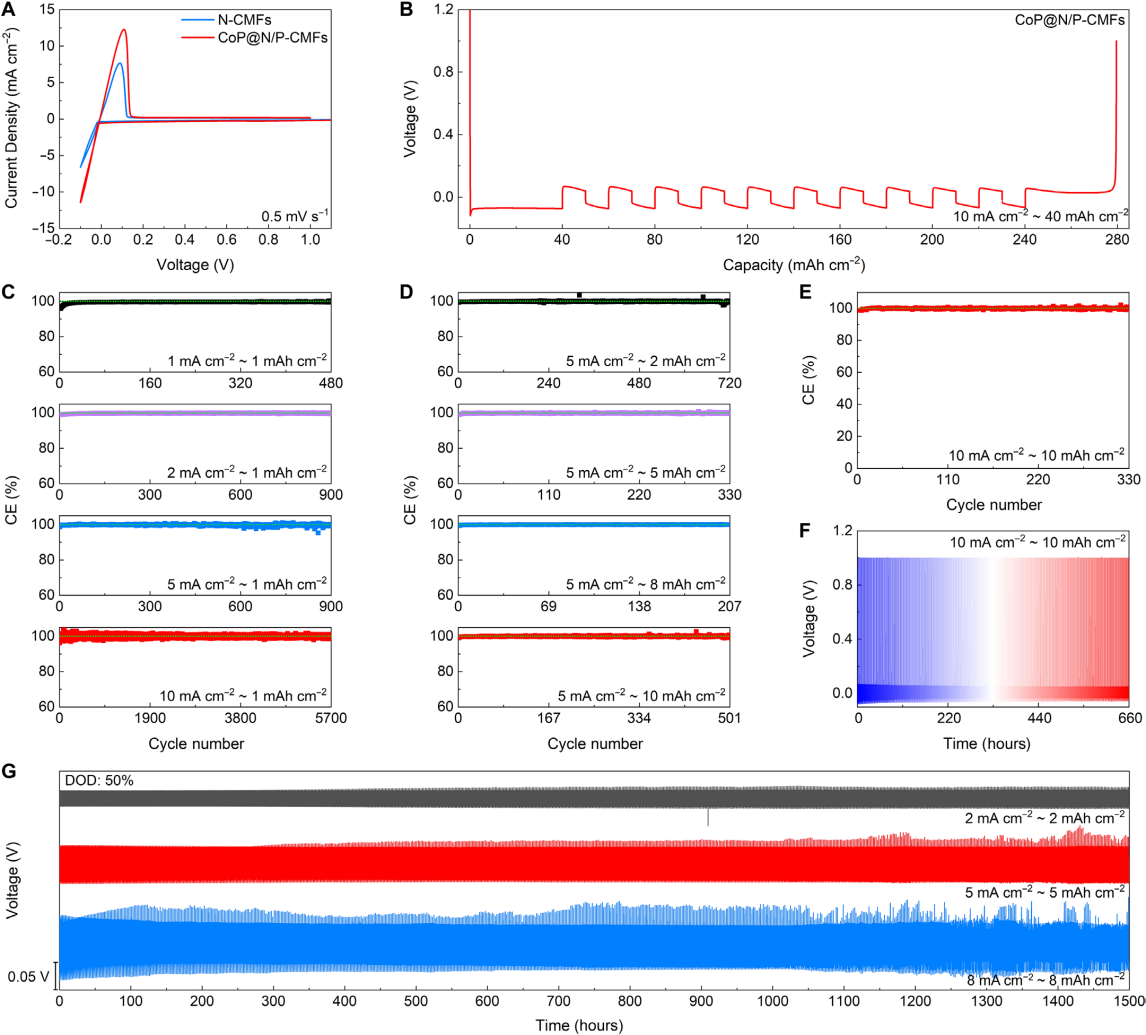
Electrochemical performance of the CoP@N/P-CMFs host. (**A**) CV profiles of different hosts tested at a scan rate of 0.5 mV s^−1^. (**B**) Voltage-capacity profiles of CoP@N/P-CMFs tested at 10 mA cm^−2^ and 40 mAh cm^−2^. (**C** and **D**) CE plots of CoP@N/P-CMFs tested at various (C) current densities and (D) areal capacities. (**E**) CE plot and (**F**) voltage-time curve of CoP@N/P-CMFs tested at 10 mA cm^−2^ and 10 mAh cm^−2^. (**G**) Cycling stability of CoP@N/P-CMFs-Na tested at various current densities and areal capacities.

To investigate Na deposition and dissolution efficiencies, which are crucial for functionality in an anode-free configuration, asymmetric cells are assembled and tested under different current densities and areal capacities. The onset potential of Na plating and stripping on the CoP@N/P-CMFs host is lower than that of the N-CMFs (Fig. 5A and fig. S26). Besides, the high current density and large closed area indicate a greater abundance of active nucleation sites in the CoP@N/P-CMFs host, further confirming rapid reaction kinetics (*49*). The CEs during the initial Na deposition and dissolution process are compared (fig. S27). The CoP@N/P-CMFs host shows high CEs compared to the N-CMFs. As the current density increases, the CoP@N/P-CMFs host exhibits higher CEs. Benefiting from its 3D structure and abundant high-affinity Na binding sites, the CoP@N/P-CMFs host achieves a high areal capacity of 40 mAh cm^−2^ with a CE of 99.61% at 10 mA cm^−2^ (Fig. 5B). Besides, electrochemical impedance spectroscopy (EIS) results indicate an obvious decrease in resistance for the CoP@N/P-CMFs (fig. S28). The effectiveness of CoP@N/P-CMFs is verified through CE tests at different current densities and areal capacities. At a fixed capacity of 1 mAh cm^−2^, the CoP@N/P-CMFs host realizes high average CEs of 99.59, 99.86, 99.84, and 99.98% at 1, 2, 5, and 10 mA cm^−2^, respectively (Fig. 5C and table S1). The CE is relatively low at a low current density, which may be ascribed to the 3D macroporous structure possibly promoting side reactions at lower current densities. Besides, the CEs of the CoP@N/P-CMFs tested at different areal capacities ranging from 2 to 10 mAh cm^−2^ are explored at 5 mA cm^−2^. The average CEs are approximately 99.93, 99.93, 99.97, and 99.96% (Fig. 5D). The corresponding charge/discharge curves for cells with CoP@N/P-CMFs are illustrated (fig. S29). Impressively, the CoP@N/P-CMFs achieve a long cycling life of more than 2000 hours at 5 mA cm^−2^ and 10 mAh cm^−2^. FESEM images reveal a dense surface for the CoP@N/P-CMFs host after cycling (fig. S30). To assess the stability of Na deposition, the Na plating current density and areal capacity are improved to 10 mA cm^−2^ and 10 mAh cm^−2^ (Fig. 5, E and F). Compared to that of the N-CMFs (fig. S31), high electrode reversibility (~99.97%), stable voltage curves, and a long cycling life (~670 hours) are obtained for the CoP@N/P-CMFs host, which is vital for constructing anode-free cells with high rate and long lifespan. The high CE of CoP@N/P-CMFs surpasses that of most hosts reported previously (table S2) (*15*, *17*, *19*–*21*, *24*, *28*, *36*, *50*–*66*).

In addition, the cycling stability of the CoP@N/P-CMFs electrode with predeposited Na (CoP@N/P-CMFs-Na) is explored at various current densities and areal capacities, under a depth-of-discharge (DOD) value of 50% (Fig. 5G and table S3). The CoP@N/P-CMFs-Na anode shows small voltage polarizations of around 30.1, 59.4, and 66.9 mV at current densities of 2, 5, and 8 mA cm^−2^ (fig. S32), respectively. At a high current density of 10 mA cm^−2^, a small voltage polarization (~72.5 mV) and a long cycling life (~700 hours) can still be achieved at a high areal capacity of 10 mAh cm^−2^ (fig. S33). In comparison, the N-CMFs host exhibits rapid cell failure (fig. S34). Besides, the cycling performance of the CoP@N/P-CMFs-Na anode at high DOD values (60 and 70%) is evaluated (fig. S35). The CoP@N/P-CMFs-Na anode delivers good durability at high DOD values. The cycling property of CoP@N/P-CMFs-Na is comparable to that of the majority of composite Na anodes on different hosts reported previously (table S4) (*15*, *18*–*21*, *24*, *25*, *28*, *50*, *54*, *56*–*67*). These results further confirm that CoP@N/P-CMFs can effectively enhance the reversibility of Na deposition and dissolution.

**Fig. 6.**
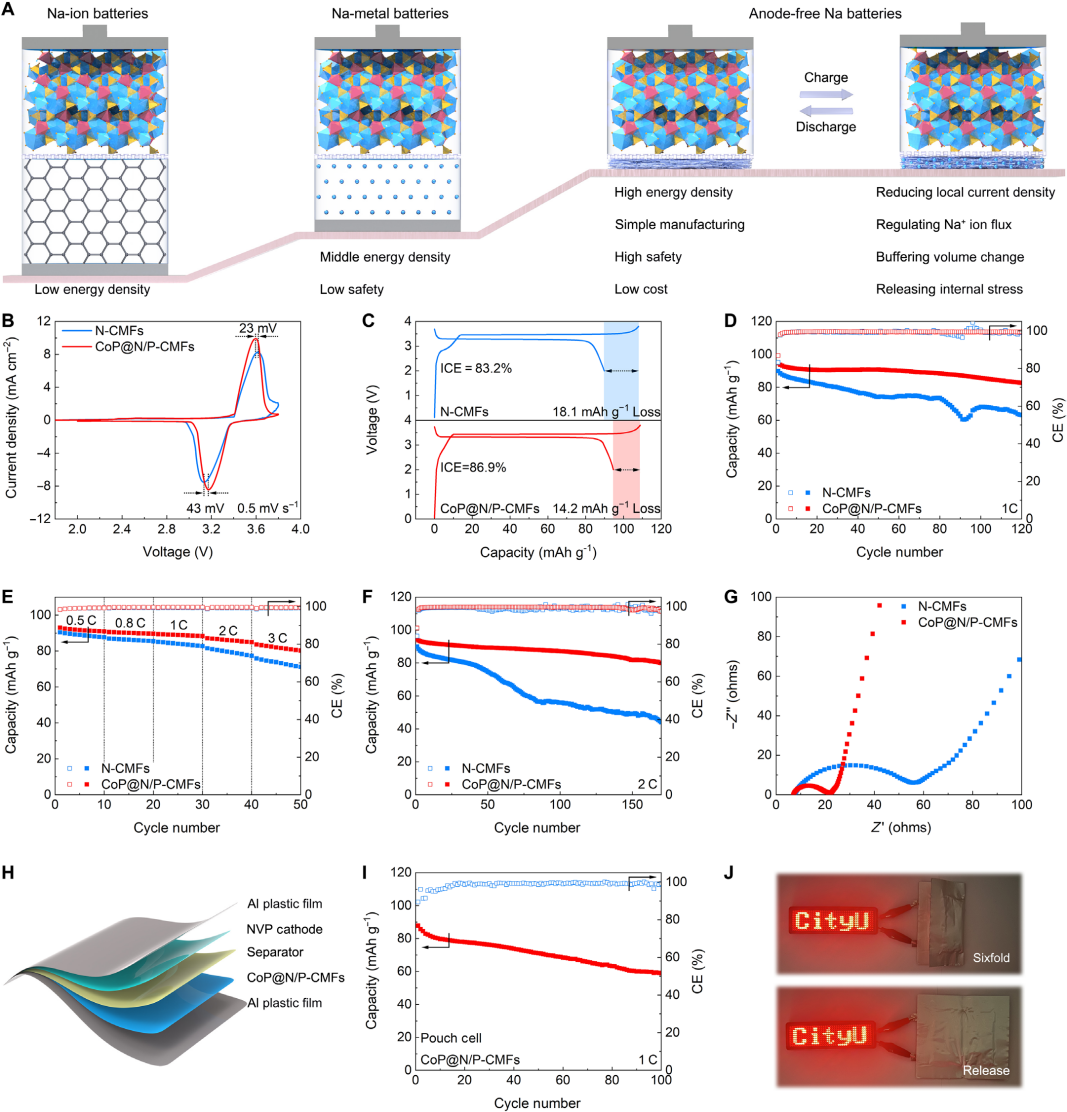
Electrochemical performance of anode-free cells. (**A**) Schematic illustration of Na-ion batteries, Na-metal batteries, and AFNBs. (**B**) CV profiles tested at a scan rate of 0.5 mV s^−1^, (**C**) first charge/discharge voltage curves, (**D**) cycling performance tested at 1 C, (**E**) rate capability, (**F**) cycling performance tested at 2 C, and (**G**) Nyquist plots of the CoP@N/P-CMFs//NVP and N-CMFs//NVP cells. (**H**) Schematic illustration and (**I**) cycling performance of the single-layer CoP@N/P-CMFs//NVP pouch cell. (**J**) Photos of the flexible CoP@N/P-CMFs//NVP pouch cell powering LEDs under various mechanical deformations.

The high reversibility of the CoP@N/P-CMFs host enables the construction of AFNBs. This anode-free configuration maximizes the energy density of the batteries (Fig. 6A). The AFNBs are assembled using NVP as the cathode (figs. S36 and S37) and N-CMFs or CoP@N/P-CMFs as the anodic current collectors. The electrochemical performance of the Na//NVP cell is measured (fig. S38). Compared to the N-CMFs//NVP cell, the CoP@N/P-CMFs//NVP cell exhibits reduced voltage polarization in CV profiles (Fig. 6B and fig. S39), indicating enhanced reaction kinetics. As displayed in Fig. 6C, the CoP@N/P-CMFs//NVP cell displays a higher initial CE of 86.9%, compared to 83.2% for the N-CMFs//NVP cell. Besides, the CoP@N/P-CMFs//NVP cell delivers more overlapped curves and higher capacity than the N-CMFs//NVP cell (fig. S40). The specific cyclability at 1 C is illustrated in Fig. 6D. The CoP@N/P-CMFs//NVP cell displays good cycling performance with a capacity retention of 87.38% after 120 cycles. In comparison, the N-CMFs//NVP cell maintains only 70.32% of its initial capacity, highlighting the effectiveness of CoP@N/P-CMFs. The rate capabilities of the N-CMFs//NVP and CoP@N/P-CMFs//NVP cells are compared in Fig. 6E, where the CoP@N/P-CMFs//NVP cell displays higher capacity at different current densities ranging from 0.5 to 3 C (fig. S41), indicating enhanced rate capability. Besides, the cycling performance of both cells at 2 C is tested (Fig. 6F and fig. S42). After 170 cycles, the CoP@N/P-CMFs//NVP cell presents a capacity of 79.88 mAh g^−1^ with a capacity retention of 84.63%. In contrast, the N-CMFs//NVP cell experiences rapid capacity decay and obvious CE fluctuations with only 44.03 mAh g^−1^ kept. EIS results indicate that the CoP@N/P-CMFs//NVP cell exhibits low charge-transfer resistance compared to the N-CMFs//NVP cell (Fig. 6G and fig. S43), which could account for the enhanced rate performance. The improved cycling property of the CoP@N/P-CMFs//NVP cell, with a high mass loading of active material in the NVP cathode, surpasses that of most previously reported AFNBs (table S5) (*14*, *15*, *17*–*21*, *24*, *25*, *28*–*30*, *50*–*53*, *55*, *67*). Inspired by the superior performance in coin cells, a pouch cell is assembled to explore the practical application of the CoP@N/P-CMFs//NVP cell (Fig. 6H). The pouch cell exhibits a capacity of 58.94 mAh g^−1^ after 100 cycles at 1 C (based on the mass of the cathode material), corresponding to a capacity retention of 67.1% (Fig. 6I). Besides, the assembled pouch cell can easily power LEDs (fig. S44). The pouch cell continues to function effectively in both its folded and released states. Unexpectedly, even under sixfold (180° × 6 deformation) conditions, the pouch cell still works well (Fig. 6J), implying the excellent flexibility and mechanical stability of CoP@N/P-CMFs.

## DISCUSSION

In summary, CoP@N/P-CMFs are reported as a 3D multifunctional current collector to achieve high-energy AFNBs. The 3D macroporous skeleton, characterized by a hollow interior and abundant void space, not only decreases local current density and regulates Na^+^ ion flux to homogenize Na deposition but also buffers volume change to achieve high Na plating capacity. From combinatorial theoretical simulations and characterization analysis, CoP nanoparticles and N-doped carbon with abundant high-affinity Na binding sites decrease Na nucleation overpotential and subsequently manipulate homogeneous Na nucleation and growth. As a result, the CoP@N/P-CMFs achieve highly reversible Na deposition and stripping with low-voltage hysteresis and an ultrahigh CE of 99.97% at 10 mA cm^−2^ and 10 mAh cm^−2^. When coupled with an NVP cathode, a foldable anode-free pouch cell is constructed, showing improved cycling performance and good rate capability. Our anode-free design offers another approach for improving the energy density of Na batteries and might be extended to other battery systems such as Li, K, Zn, Mg, and Al. Thus far, improving cycling performance is essential to facilitate the transition from laboratory-scale to industrial-scale applications. This may be realized by designing artificial SEI to reduce the losses of active Na ions, developing Na-rich cathodes to improve the availability of active Na ions, and engineering anodic current collector to improve the cycling life of active Na ions.

## MATERIALS AND METHODS

### Synthesis of ZIF-67 NCs

In a typical procedure, 876 mg of Co(NO_3_)_2_·6H_2_O was dissolved in 30 ml of DIW with a certain amount of CTAB ranging from 12 to 24 mg. This solution was then rapidly added to 210 ml of an aqueous solution containing 13.62 g of 2-methylimidazole and stirred at ambient temperature for 60 min. Last, the precipitate was gathered by centrifugation and washed with ethanol.

### Synthesis of PA-Co NBs

The ZIF-67 NCs were dispersed in 150 ml of an ethanol/DIW solution. This mixture was then added to another 150 ml of an ethanol/DIW solution containing 8 mmol PA, with the water content varying from 0 to 16 ml. After stirring for 10 min, the precipitate was collected by centrifugation and washed with ethanol.

### Synthesis of CoP@N/P-CMFs

The as-prepared PA-Co NBs (260 mg) were dispersed in 0.8 ml of dimethylformamide (DMF) and mixed with 80 mg of PAN (molecular weight ~150,000) under stirring for 12 hours at 60°C to obtain the precursor solution. The PA-Co@PAN was then electrospun onto Al foil using this precursor solution, with a feeding rate of 1.0 ml h^−1^ and a high voltage of 20 kV. The distance between the stainless-steel needle and the collector was set at 12 cm. Subsequently, the PA-Co@PAN was stabilized at 150°C for 2 hours and then annealed in a 5% H_2_/Ar atmosphere. The calcination process was performed from ambient temperature to 400°C with a ramp rate of 2°C min^−1^ and kept for 1 hour. Afterward, the temperature was raised to 800°C at a consistent rate and maintained for 2 hours. The precarbonation process at 400°C during calcination is essential to preserve the 3D macroporous structure. During the calcination process, PAN was converted to N-doped carbon.

### Synthesis of N-CMFs

N-CMFs were fabricated using a process similar to that of CoP@N/P-CMFs, but with different precursor solutions. Specifically, 0.1 g of PAN was dissolved in 0.8 ml of DMF under stirring at 60°C for 12 hours to gain the precursor solution.

### Materials characterizations

The phase composition and structure were examined using XRD on a Bruker D2 Phaser x-ray diffractometer. The specific surface area was calculated by nitrogen adsorption isotherms tested with a Micromeritics ASAP 2460 based on the BET model. The elemental chemical states were analyzed by XPS (ESCALAB 250Xi). Raman spectra were examined on a Renishaw inVia confocal Raman microscope. The structure and morphology were investigated using FESEM (JSM-7800F) and high-resolution TEM (HRTEM, JEOL JEM-2100). Elemental mapping images were acquired by TEM equipped with EDX spectroscopy. The compositions were assessed by EDX spectroscopy attached to HRTEM and FESEM instruments.

### Electrochemical measurements

The electrochemical tests were executed by assembling 2032 coin cells on a NEWARE battery tester (CT-4008-5 V 10 mA-164). Glass fiber was used as a separator. Sodium hexafluorophosphate (NaPF_6_; 1 M) in diglyme was used as an electrolyte. To explore the CE of Na plating/stripping, N-CMFs and CoP@N/P-CMFs were used as the working electrodes, and Na foil was used as the counter/reference electrode. For the cycling tests, metallic Na (4, 10, 16, and 20 mAh cm^−2^) was preplated on N-CMFs and CoP@N/P-CMFs to obtain N-CMFs-Na and CoP@N/P-CMFs-Na electrodes tested at different current densities (2, 5, 8, and 10 mA cm^−2^) with a DOD value of 50%. For anode-free cell tests, commercial NVP materials were used as the cathode. To fabricate NVP cathode, NVP powder, polyvinylidene fluoride, and Ketjen black were mixed with a weight ratio of 94:2:4 into *N*-methyl-2-pyrrolidone solvent. Subsequently, the slurry was pasted onto an Al foil and dried overnight at 120°C under a vacuum condition. The mass loading of active material is around 18 mg cm^−2^. Full cells were assembled using NVP as the cathode and N-CMFs or CoP@N/P-CMFs as the anodic current collector.
