# Supplementary material for: Foldable anode-free sodium batteries enabled by N,P-codoped carbon macroporous fibers incorporated with CoP nanoparticles
Source: Sci Adv. 2025 May 9;11(19):eadv2007. doi: 10.1126/sciadv.adv2007 (PMC12063667; doi:10.1126/sciadv.adv2007)
Supplement: Supplementary file 1 — Supplementary Text Figs. S1 to S44 Tables S1 to S5 [file sciadv.adv2007_sm.pdf]

Science Advances
AAAS

Supplementary Materials for

Foldable anode-free sodium batteries enabled by N,P-codoped carbon macroporous fibers incorporated with CoP nanoparticles

Yongling An *et al.*

Corresponding author: Xiong Wen (David) Lou, david.lou@cityu.edu.hk

Sci. Adv. **11**, eadv2007 (2025)
DOI: 10.1126/sciadv.adv2007

This PDF file includes:

Supplementary Text
Figs. S1 to S44
Tables S1 to S5

1. Supplementary Text

Computational details

Vienna ab initio simulation package (VASP) was utilized to conduct the density functional theory (DFT) calculation. The ion-electron interactions were characterized using the projector augmented wave (PAW) approach. Generalized gradient approximation (GGA) in the scheme of Perdew-Burke-Ernzerhof (PBE) was employed to model electron exchange and correlation interactions. Plane-wave basis with wave function cut-off energy of 450 eV was employed. The atomic relaxation was halted once the total energy tolerance reached 10^{-5} eV and the changes of the force on atoms were below 0.02 eV $Å^{-1}$. Additionally, all structures were optimized using a Monkhorst-Pack K-point grid of (2×2×1) to account for the symmetry of supercell, cost of used time, and accuracy of calculation.

2. Figures in the Supplementary Materials.

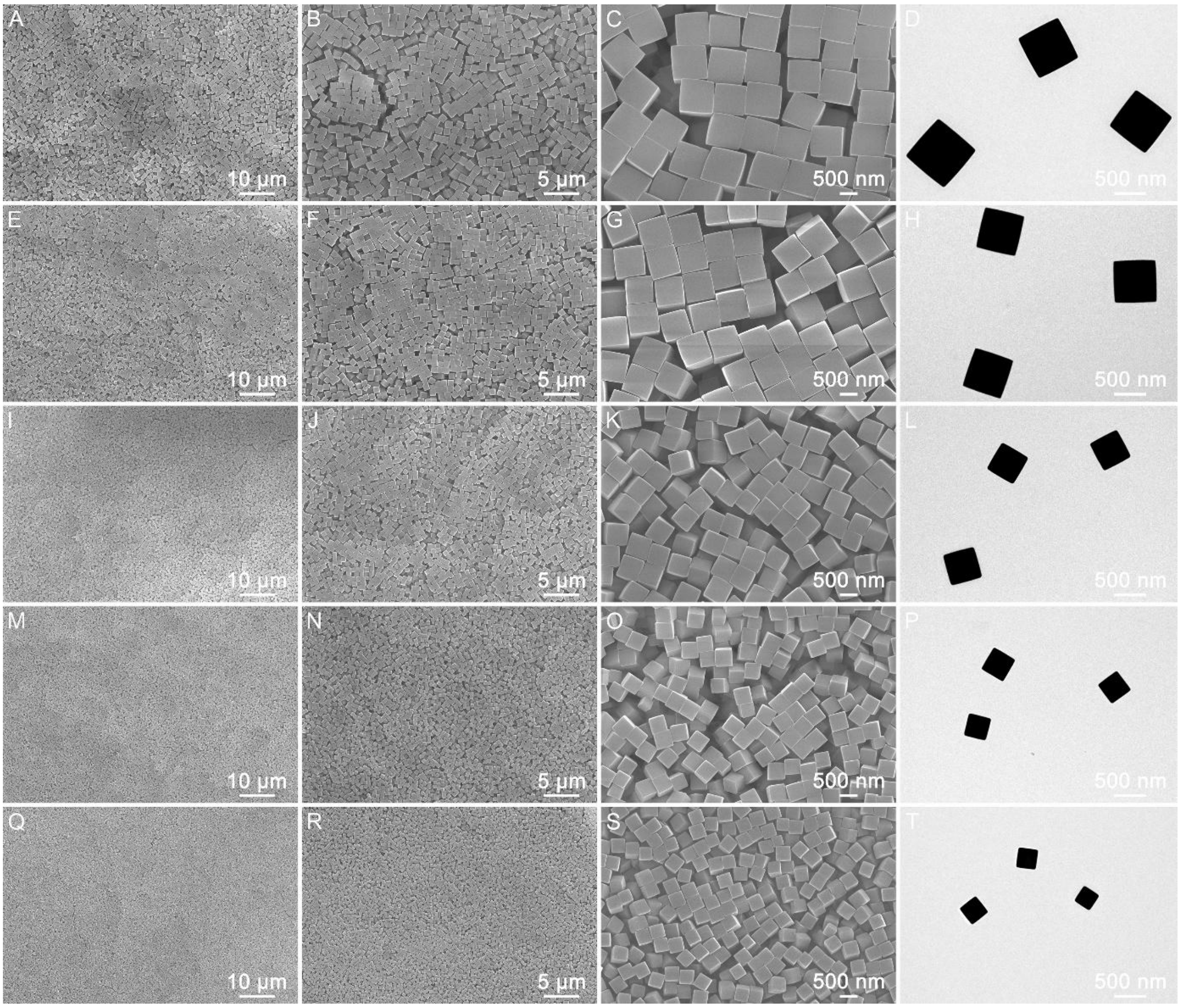

Fig. S1. FESEM and TEM measurements. (**A** to **C**, **E** to **G**, **I** to **K**, **M** to **O**, **Q** to **S**) FESEM and (**D**, **H**, **L**, **P**, **T**) TEM images of ZIF-67 NCs obtained with different CTAB amounts. (**A** to **D**) 12 mg, (**E** to **H**) 15 mg, (**I** to **L**) 18 mg, (**M** to **P**) 21 mg, and (**Q** to **T**) 24 mg.

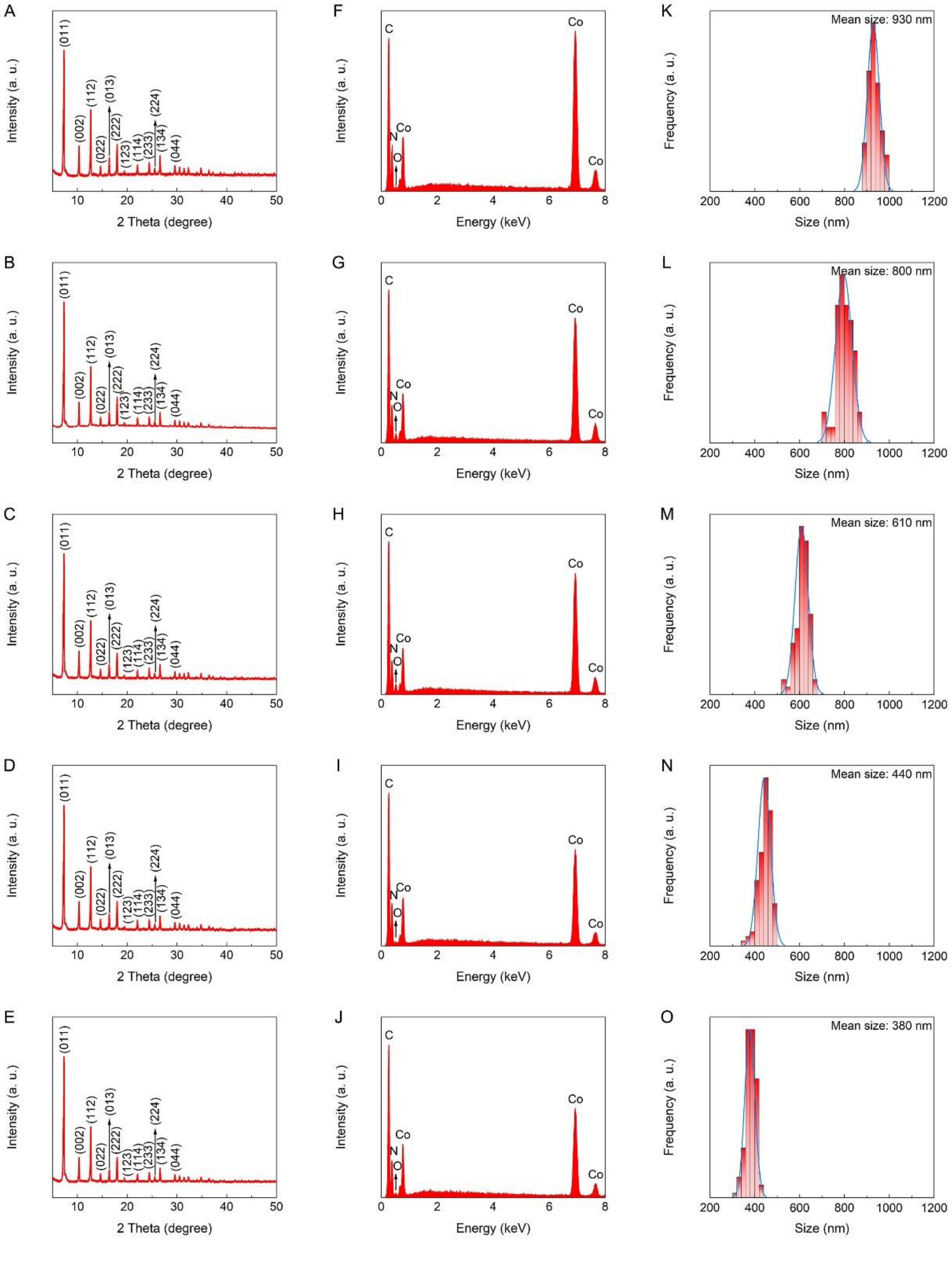

Fig. S2. XRD, EDX, and size distribution measurements. (**A** to **E**) XRD patterns, (**F** to **J**) EDX spectra, and (**K** to **O**) size distributions of ZIF-67 NCs obtained with different CTAB amounts. (**A**, **F**, **K**) 12 mg, (**B**, **G**, **L**) 15 mg, (**C**, **H**, **M**) 18 mg, (**D**, **I**, **N**) 21 mg, and (**E**, **J**, **O**) 24 mg.

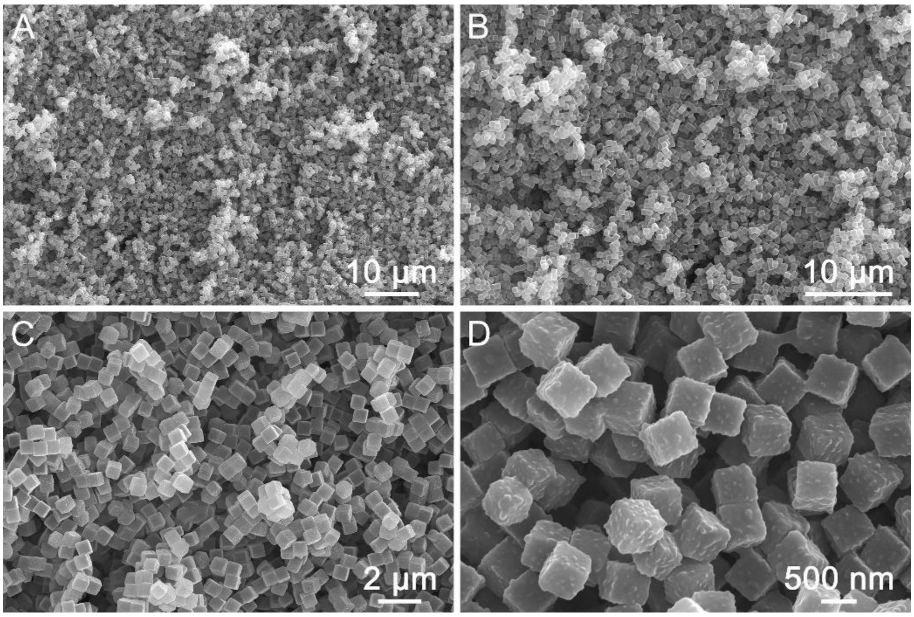

Fig. S3. FESEM measurement. FESEM images of PA-Co NBs.

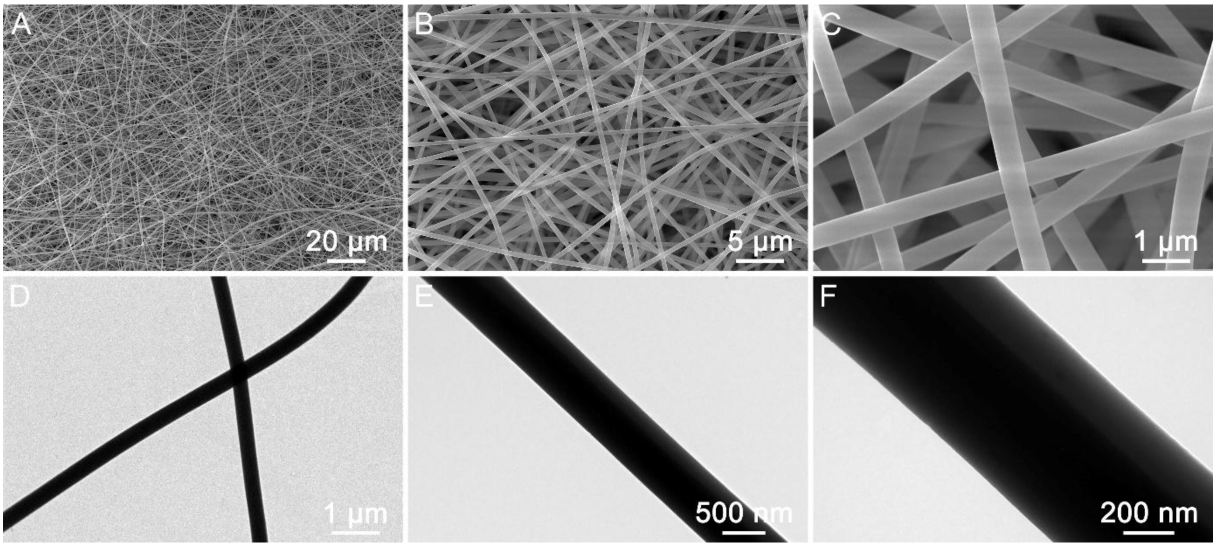

Fig. S4. FESEM and TEM measurements. (**A** to **C**) FESEM and (**D** to **F**) TEM images of PAN.

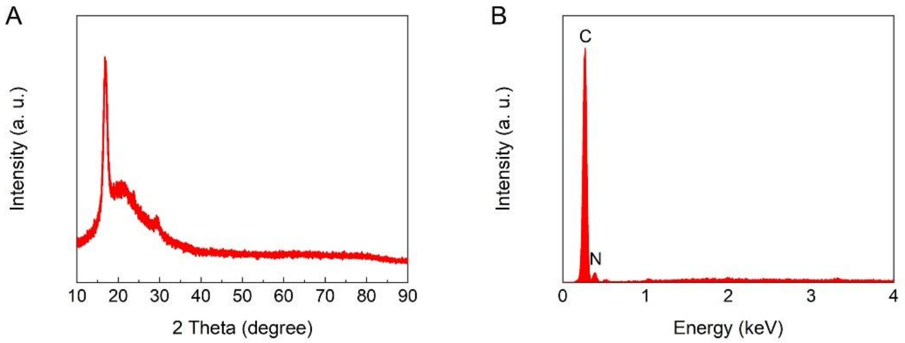

Fig. S5. XRD and EDX measurements. (**A**) XRD pattern and (**B**) EDX spectrum of PAN.

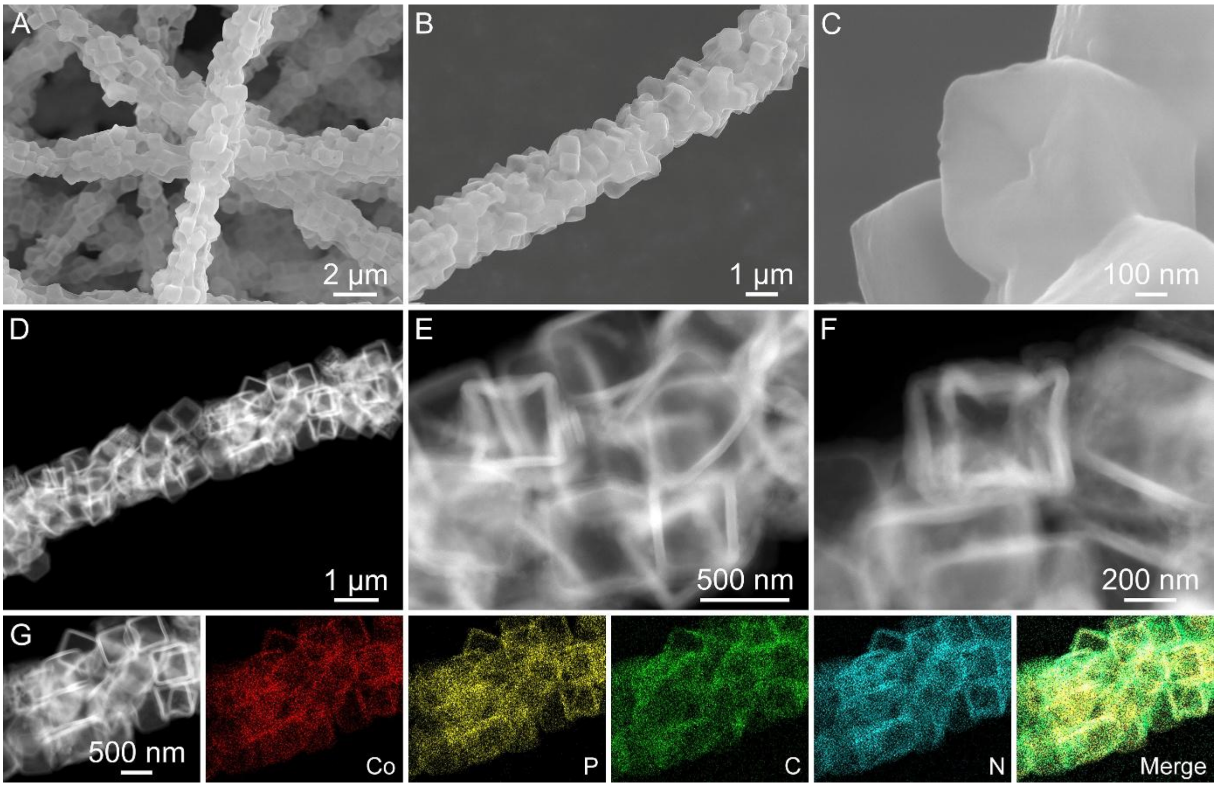

Fig. S6. FESEM and TEM measurements. (**A** to **C**) FESEM, (**D** to **F**) HAADF-STEM, and (**G**) elemental mapping images of PA-Co@PAN.

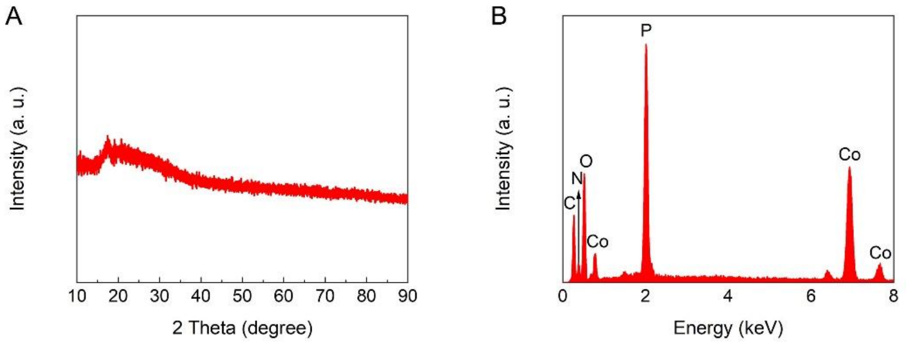

Fig. S7. XRD and EDX measurements. (**A**) XRD pattern and (**B**) EDX spectrum of PA-Co@PAN.

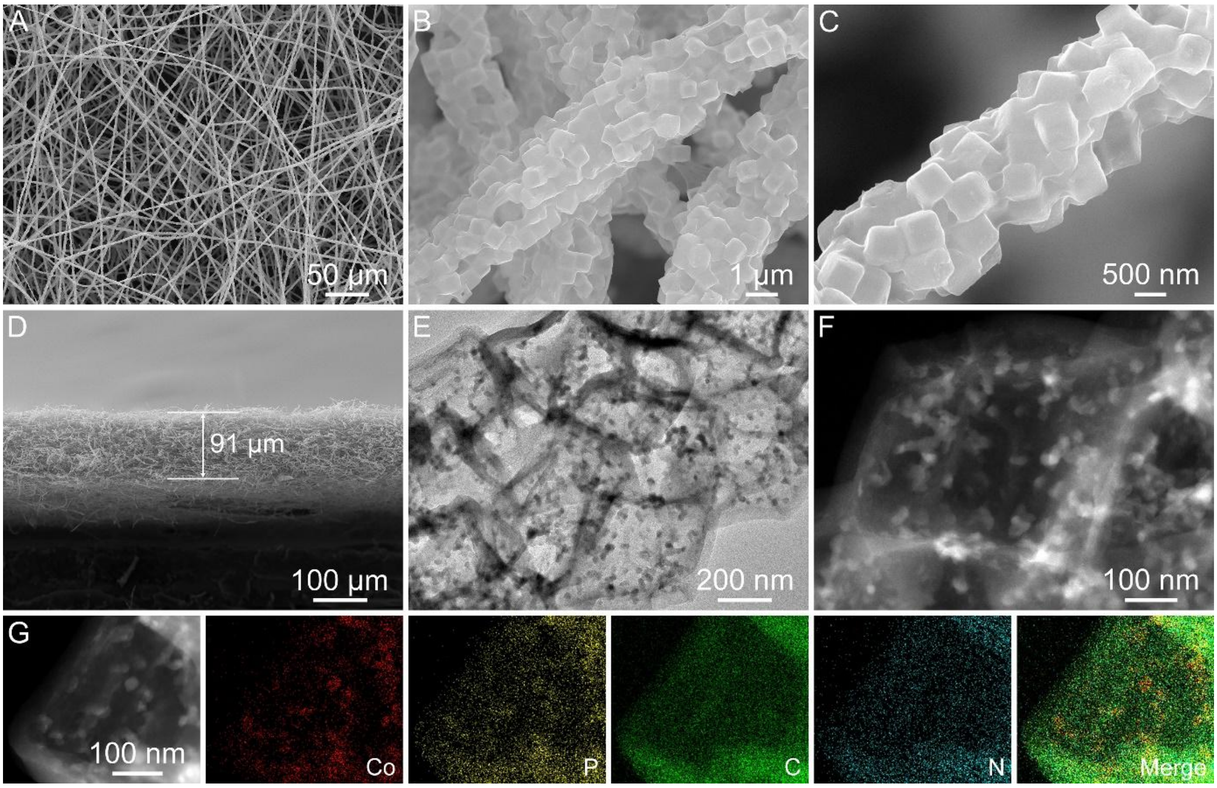

Fig. S8. FESEM and TEM measurements. (**A** to **C**) FESEM, (**D**) cross-sectional FESEM, (**E**) TEM, (**F**) HAADF-STEM, and (**G**) elemental mapping images of CoP@N/P-CMFs.

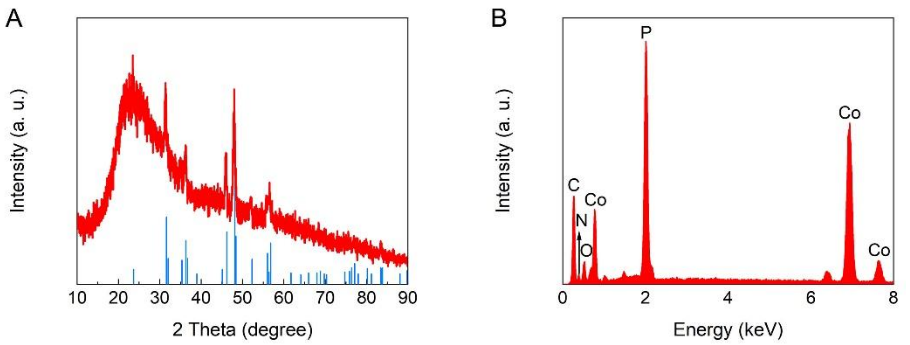

Fig. S9. XRD and EDX measurements. (**A**) XRD pattern and (**B**) EDX spectrum of CoP@N/P-CMFs.

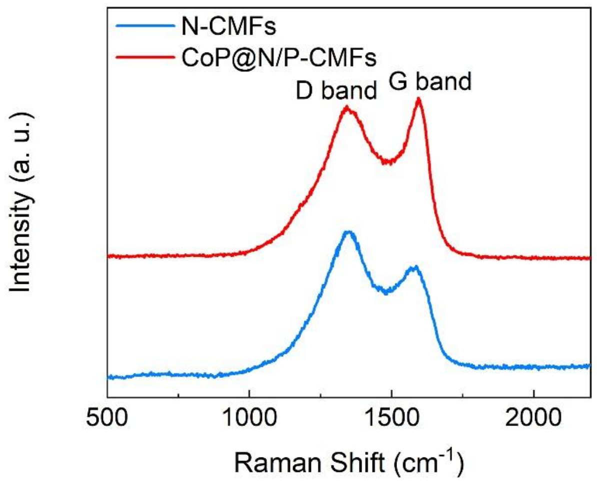

Fig. S10. Raman measurement. Raman spectra of N-CMF and CoP@N/P-CMFs.

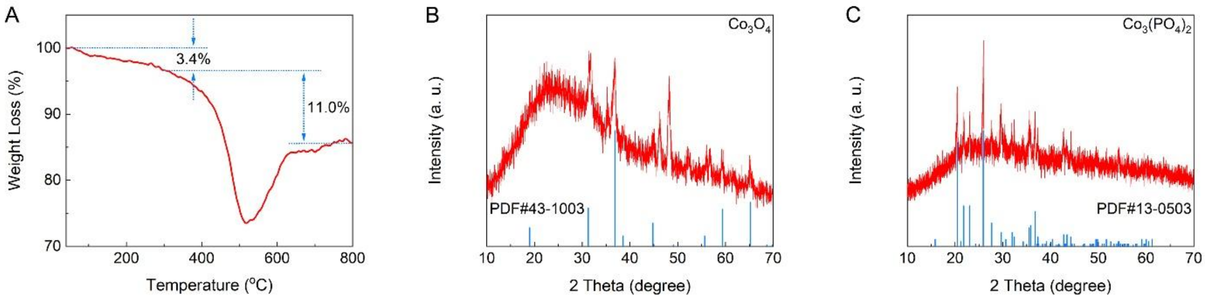

Fig. S11. TGA and XRD measurements. (**A**) TGA curve of CoP@N/P-CMFs. (**B** and **C**) XRD patterns of CoP@N/P-CMFs corresponding to the TGA curve at (B) 500 ^{o}C and (C) 800 ^{o}C.

During the TGA measurement, the CoP will undergo the following reactions (*42*),

$12CoP + 23O_2 \rightarrow 4Co_3O_4 + 6P_2O_5$

$6CoP + Co_3O_4 + 10O_2 \rightarrow 3Co_3(PO_4)_2$

Thus, the overall reaction is

$12CoP + 21O_2 \rightarrow 2P_2O_5 + 4Co_3(PO_4)_2$

CoP is converted to $Co_3(PO_4)_2$, resulting in a mass increase of 36%. Assuming the mass percentage of carbon in the CoP@N/P-CMFs is m, the mass percentage of CoP is 1-m. According to the computational formula,

m - 36% (1-m) = 11.0%

then, one can obtain m = 34.6%.

So, the carbon content in the CoP@N/P-CMFs is about 34.6%.

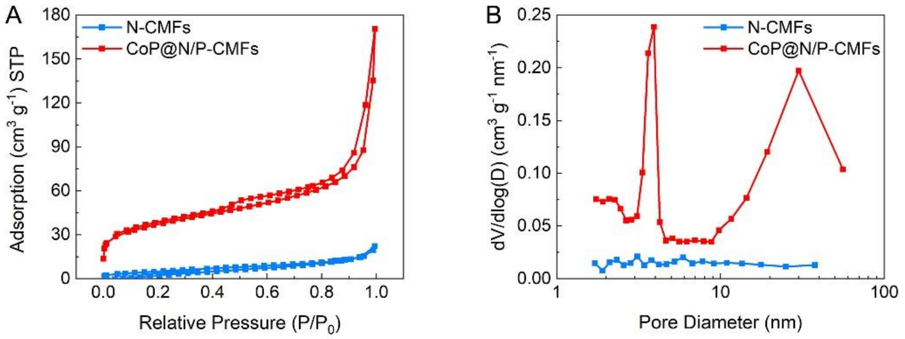

Fig. S12. BET analysis. (**A**) N_2 adsorption-desorption isotherms and (**B**) pore size distributions of N-CMF and CoP@N/P-CMFs.

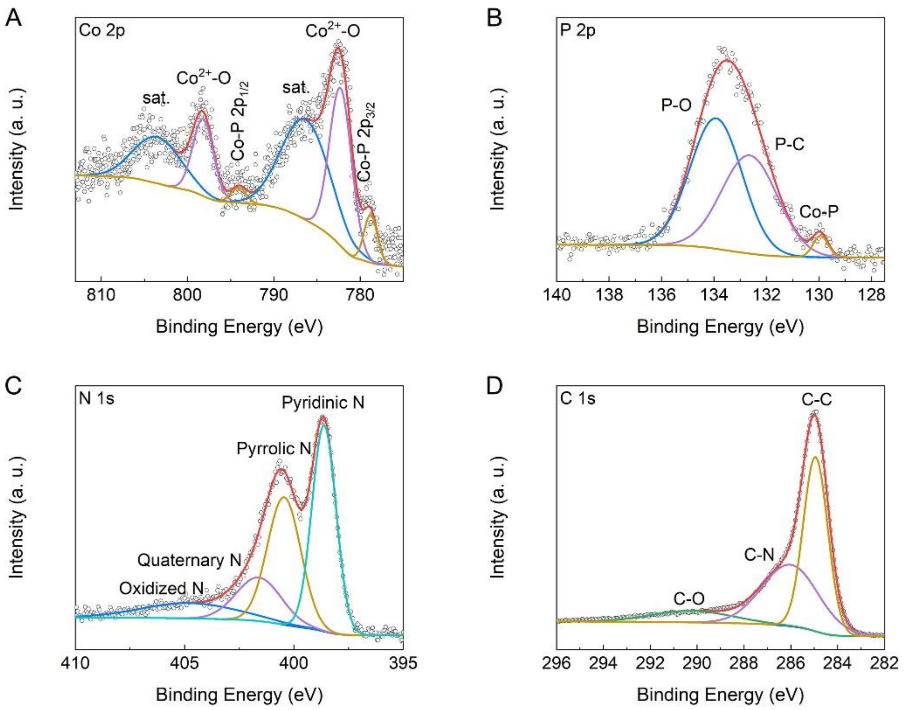

Fig. S13. XPS measurement. High-resolution (**A**) Co 2p, (**B**) P 2p, (**C**) N 1s, and (**D**) C 1s XPS spectra of CoP@N/P-CMFs.

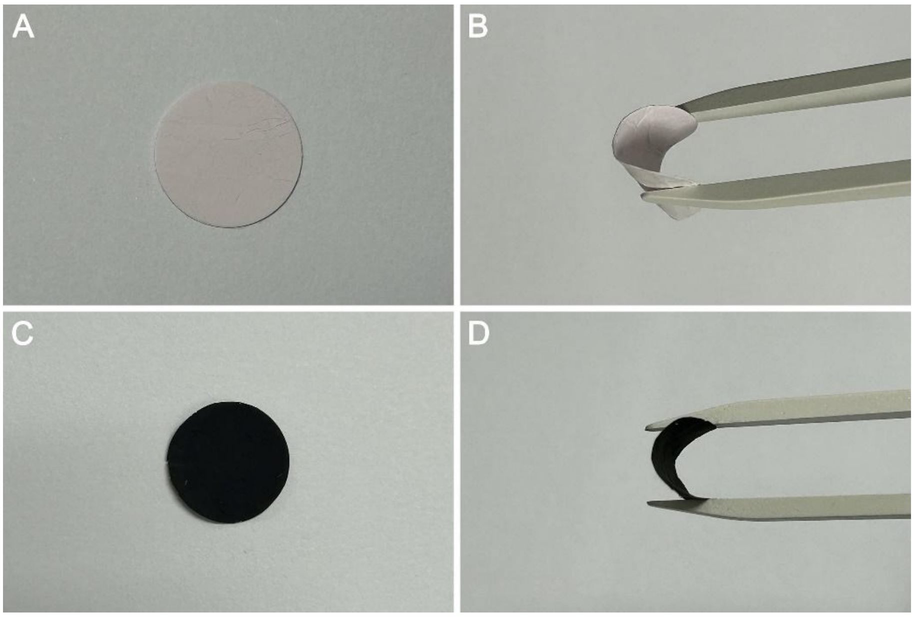

Fig. S14. Photos of (**A** and **B**) PA-Co@PAN and (**C** and **D**) CoP@N/P-CMFs.

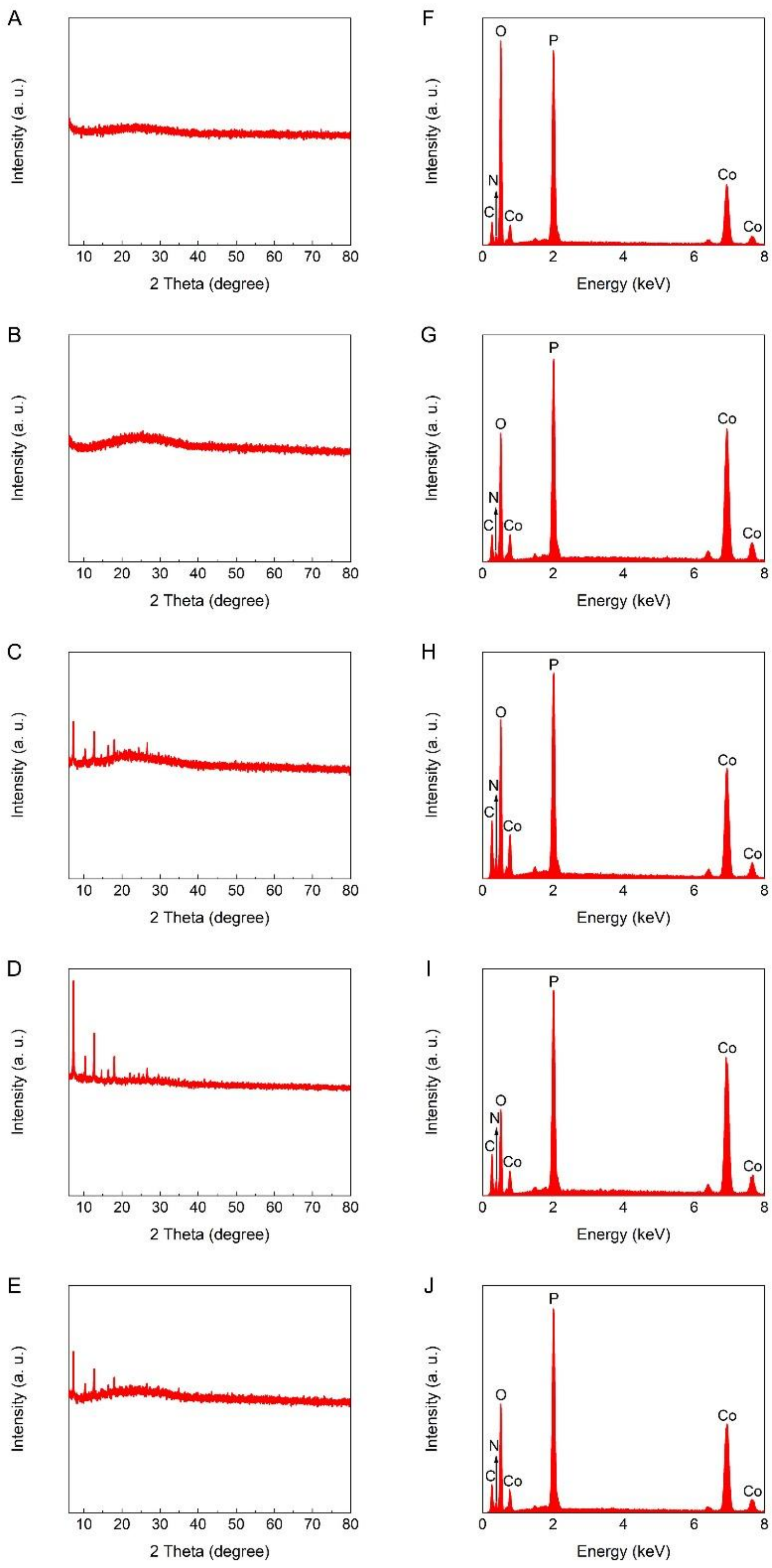

Fig. S15. XRD and EDX measurements. (**A** to **E**) XRD patterns and (**F** to **G**) EDX spectra of PA-Co obtained after etching with different DIW contents in an ethanol/DIW solution: (**A** and **F**) 0 mL, (**B** and **G**) 4 mL, (**C** and **H**) 8 mL, (**D** and **I**) 12 mL, and (**E** and **J**) 16 mL.

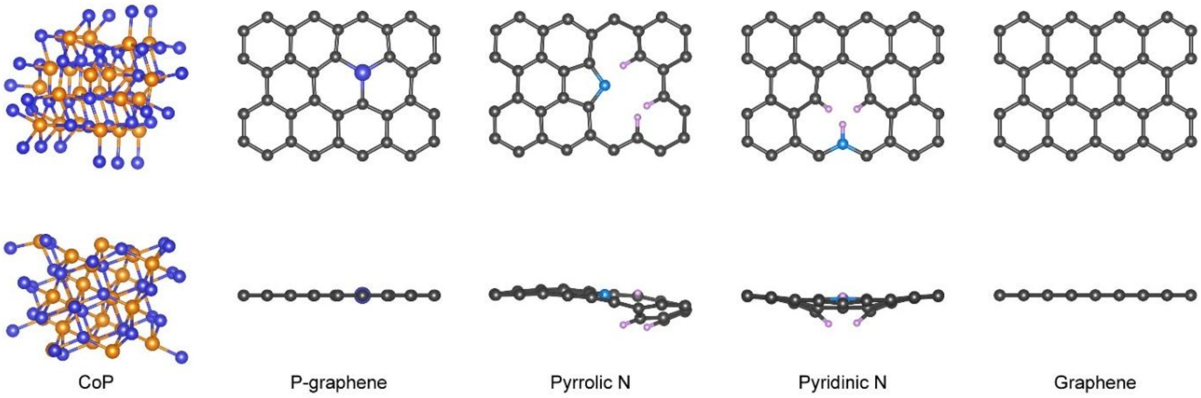

Fig. S16. Structural model. Models of CoP, P-doped graphene, pyrrolic N-doped graphene, pyridinic N-doped graphene, and graphene.

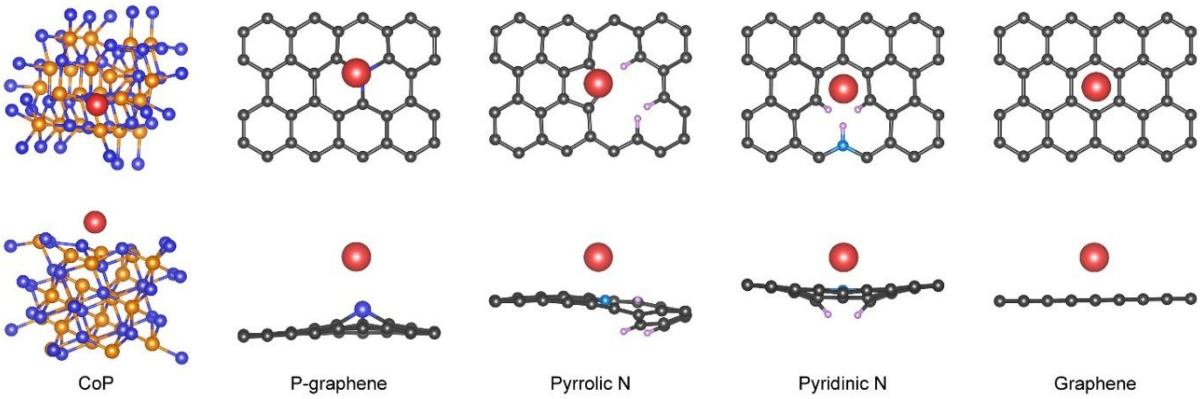

Fig. S17. Structural model. Models of CoP, P-doped graphene, pyrrolic N-doped graphene, pyridinic N-doped graphene, and graphene after Na atoms adsorption.

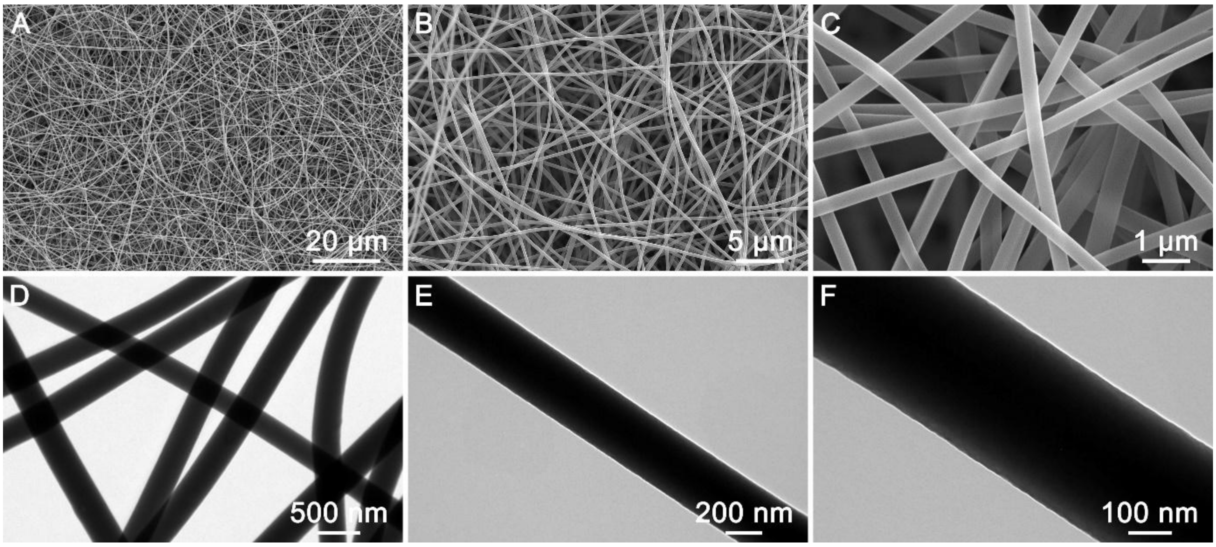

Fig. S18. FESEM and TEM measurements. (**A** to **C**) FESEM and (**D** to **F**) TEM images of N-CMFs.

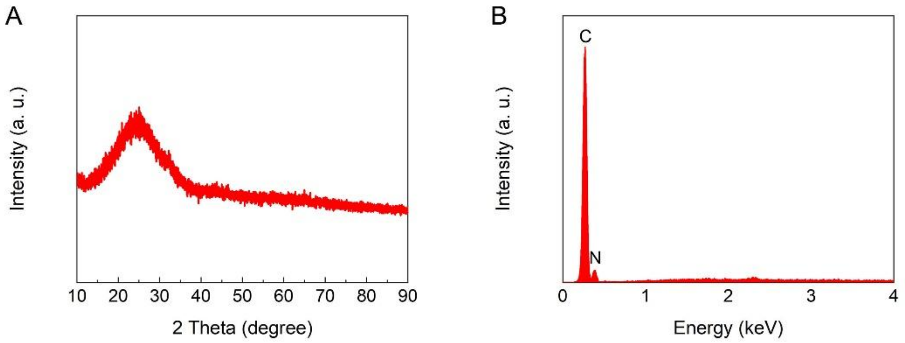

Fig. S19. XRD and EDX measurements. (**A**) XRD pattern and (**B**) EDX spectrum of N-CMFs.

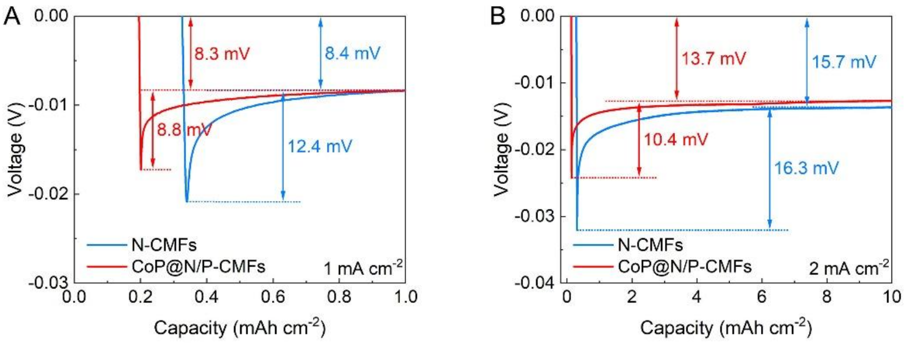

Fig. S20. Voltage-capacity analysis. Voltage-capacity profiles during Na nucleation on the N-CMFs and CoP@N/P-CMFs hosts at different current densities of (**A**) 1 mA cm^{-2} and (**B**) 2 mA cm^{-2}.

The high binding energy can effectively reduce the energy barrier of Na nucleation, further promoting the homogenous Na nucleation and growth. Compared to the N-CMFs, the CoP@N/P-CMFs with abundant sodiophilic sites exhibit higher Na affinity and stronger binding with Na, minimizing the Na nucleation overpotential and uniformizing the Na^+ flux.

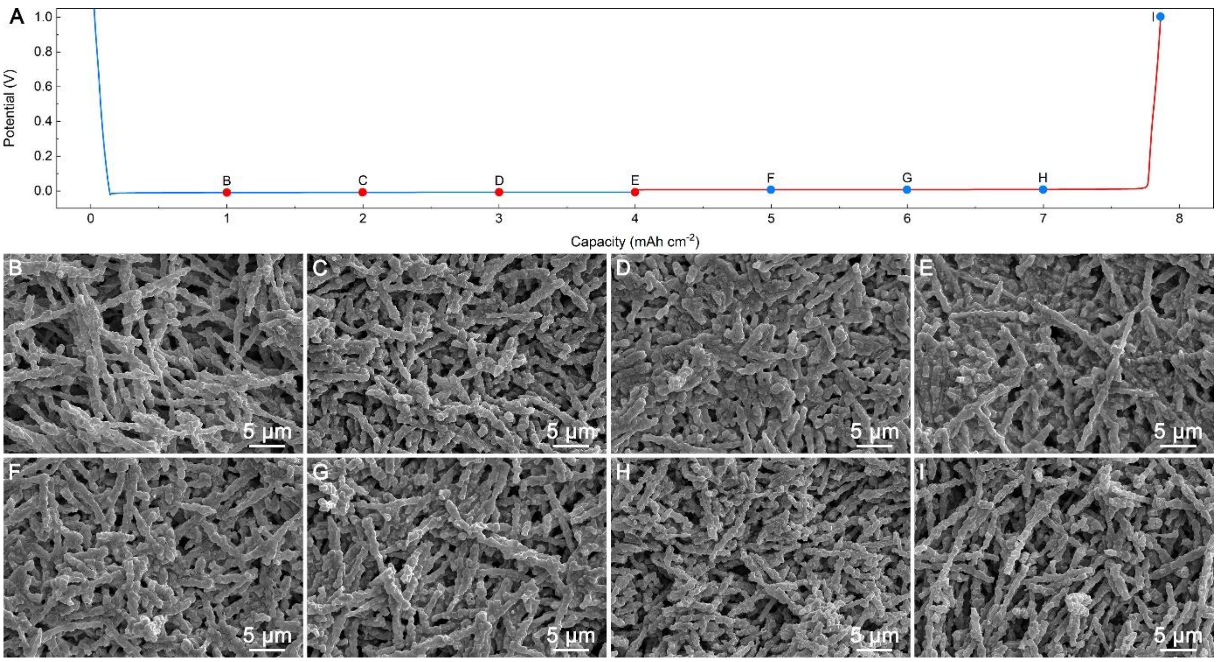

Fig. S21. Na metal deposition behavior on CoP@N/P-CMFs. (**A**) Electrochemical Na plating/stripping voltage profile of CoP@N/P-CMFs at 1 mA cm^{-2} for a capacity of 4 mAh cm^{-2}. (**B** to **E**) FESEM images of CoP@N/P-CMFs host after plating (**B**) 1 mAh cm^{-2}, (**C**) 2 mAh cm^{-2}, (**D**) 3 mAh cm^{-2}, and (**E**) 4 mAh cm^{-2} of Na metal into the CoP@N/P-CMFs. (**F** to **I**) Anodes after stripping (**F**) 1 mAh cm^{-2}, (**G**) 2 mAh cm^{-2}, (**H**) 3 mAh cm^{-2}, and (**I**) 4 mAh cm^{-2} (recharged to 1 V) from Na anodes with CoP@N/P-CMFs.

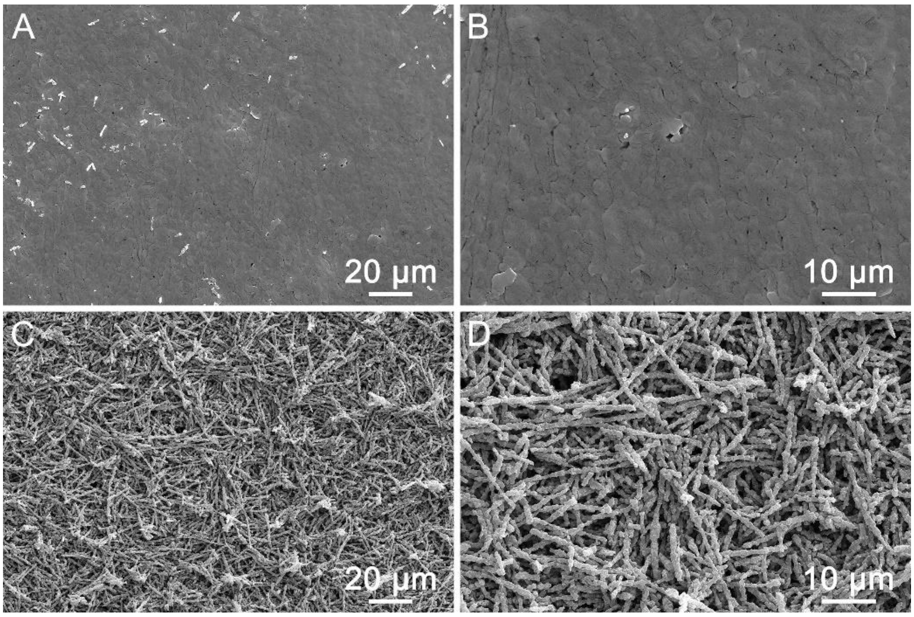

Fig. S22. FESEM measurement. FESEM images of CoP@N/P-CMFs after (**A** and **B**) Na plating with a capacity of 10 mAh cm^{-2} and (**C** and **D**) stripping to 1 V.

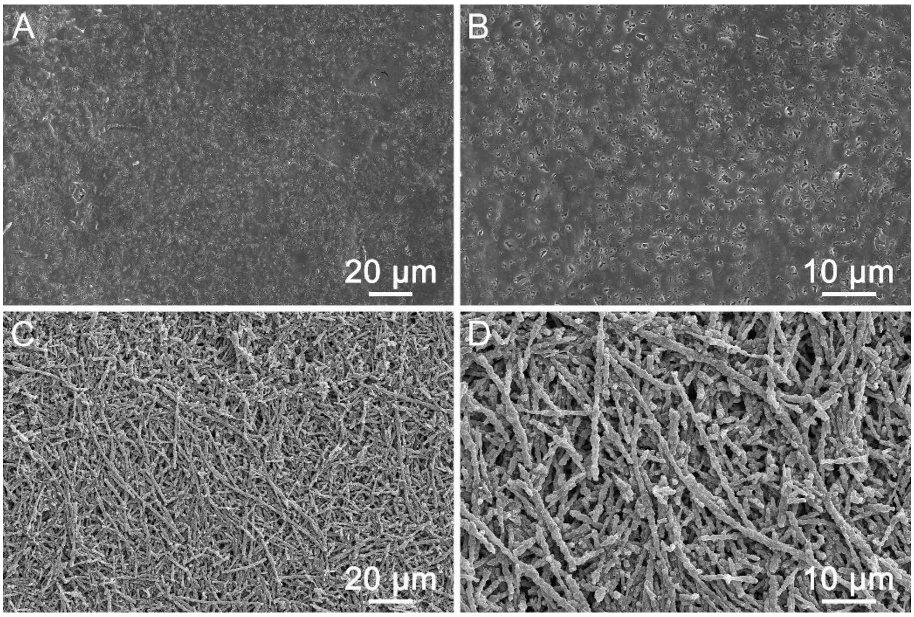

Fig. S23. FESEM measurement. FESEM images of CoP@N/P-CMFs after (**A** and **B**) Na plating with a capacity of 20 mAh cm^{-2} and (**C** and **D**) stripping to 1 V.

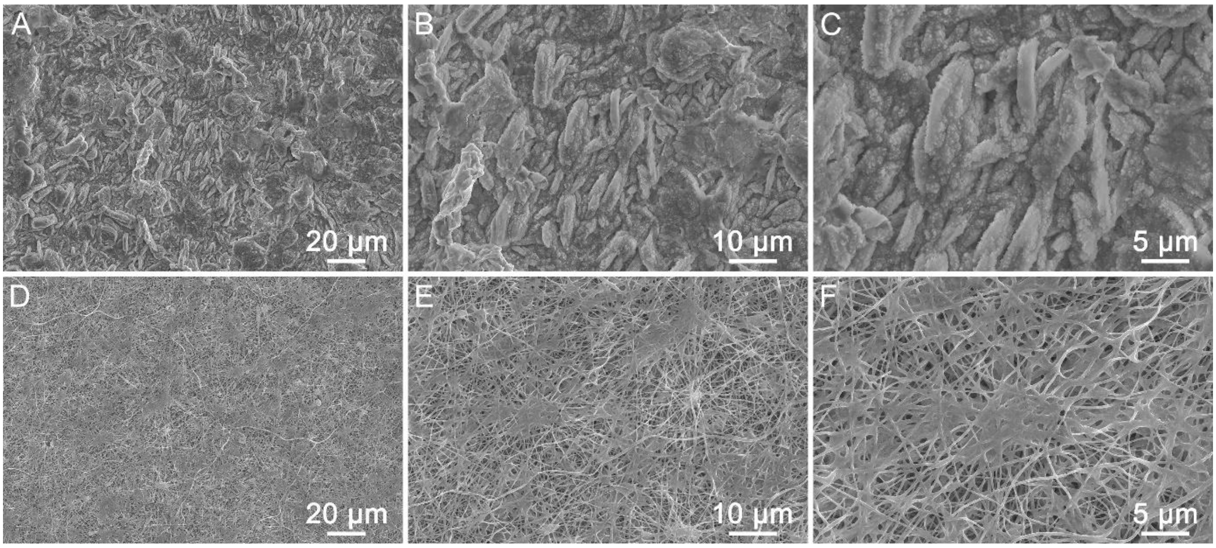

Fig. S24. FESEM measurement. FESEM images of N-CMFs after (**A** to **C**) Na plating with a capacity of 10 mAh cm^{-2} and (**D** to **F**) stripping to 1 V.

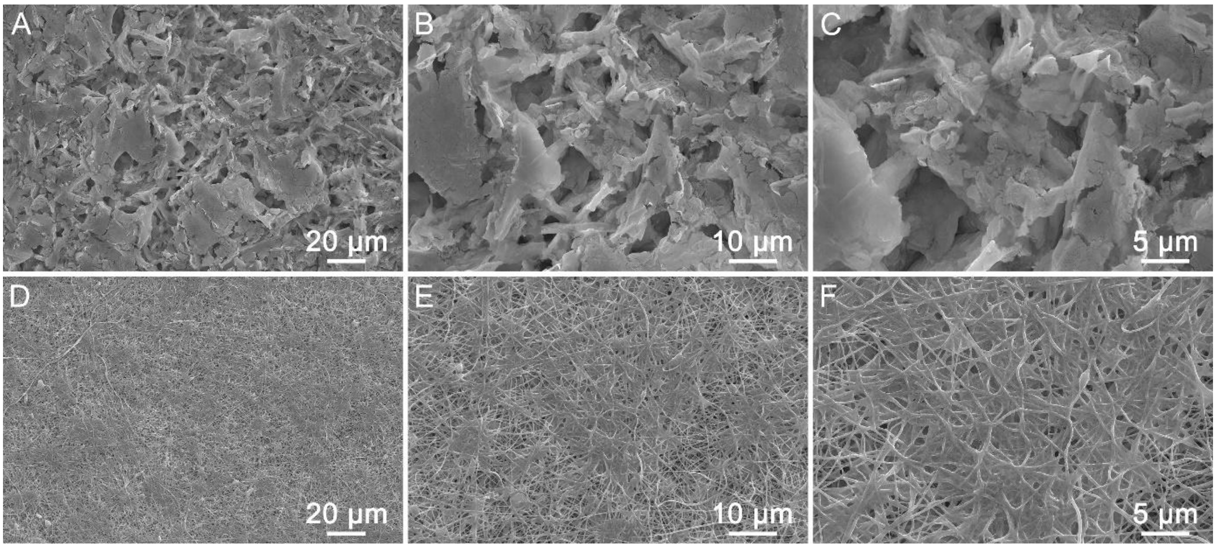

Fig. S25. FESEM measurement. FESEM images of N-CMFs after (**A** to **C**) Na plating with a capacity of 20 mAh cm^{-2} and (**D** to **F**) stripping to 1 V.

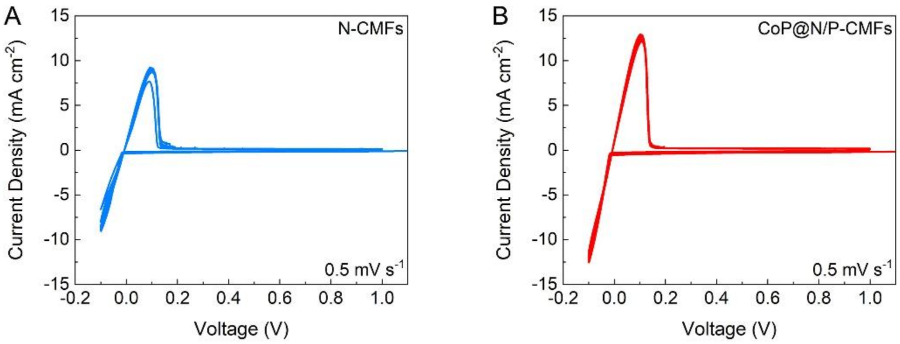

Fig. S26. CV analysis. CV curves of (**A**) N-CMFs and (**B**) CoP@N/P-CMFs electrodes at a scan rate of 0.5 mV s^{-1}.

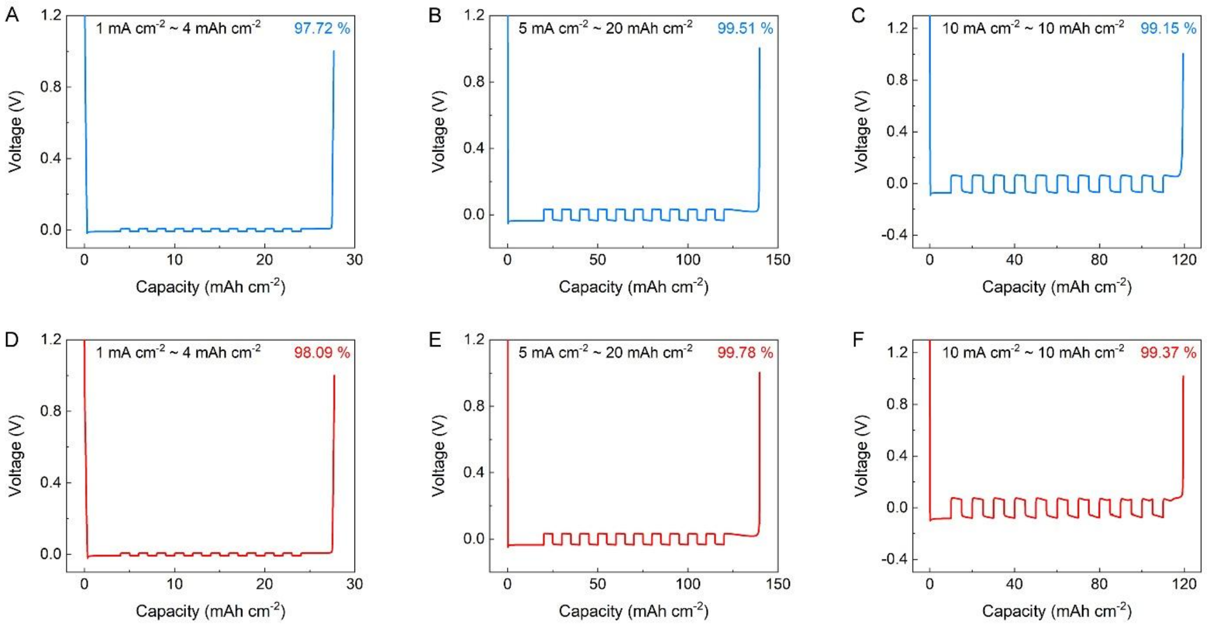

Fig. S27. CE analysis. Voltage-capacity profiles of (**A** to **C**) N-CMFs and (**D** to **F**) CoP@N/P-CMFs electrodes tested at different current densities and areal capacities: (**A** and **D**) 1 mA cm^{-2} and 4 mAh cm^{-2}, (**B** and **E**) 5 mA cm^{-2} and 20 mAh cm^{-2}, (**C** and **F**) 10 mA cm^{-2} and 10 mAh cm^{-2}.

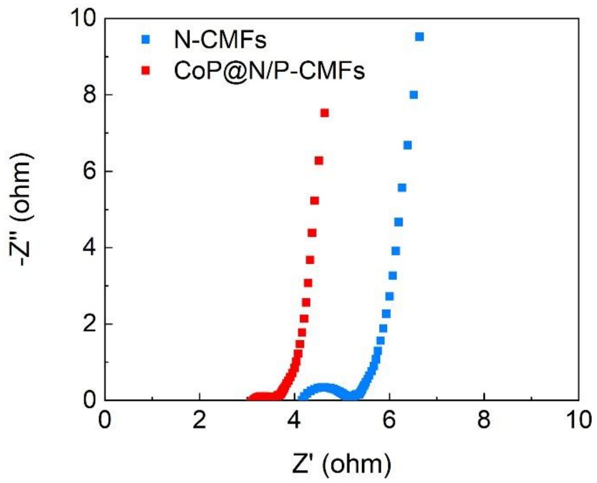

Fig. S28. EIS analysis. Nyquist plots of different hosts after initial Na plating/stripping process.

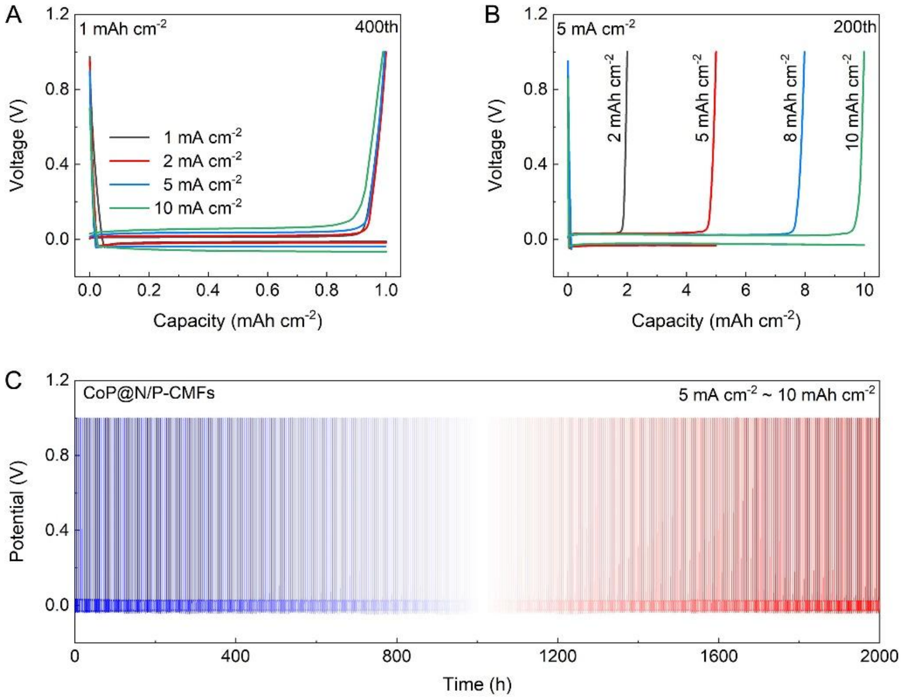

Fig. S29. CE analysis. (**A** and **B**) Voltage-capacity curves of CoP@N/P-CMFs tested at different (**A**) current densities and (**B**) areal capacities. (**C**) Voltage-time curve of CoP@N/P-CMFs tested at 5 mA cm^{-2} and 10 mAh cm^{-2}.

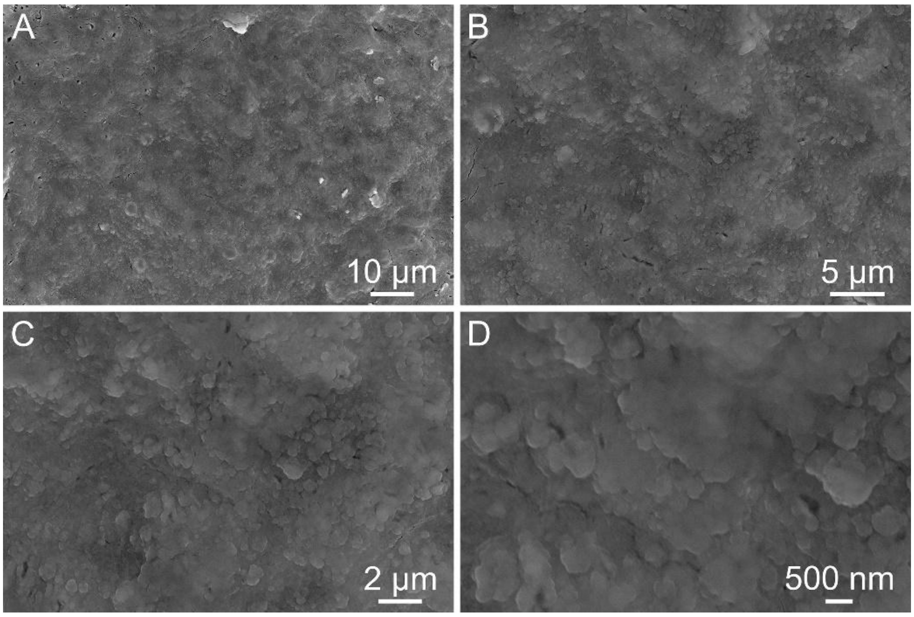

Fig. S30. FESEM measurement. FESEM images of CoP@N/P-CMFs after cycling.

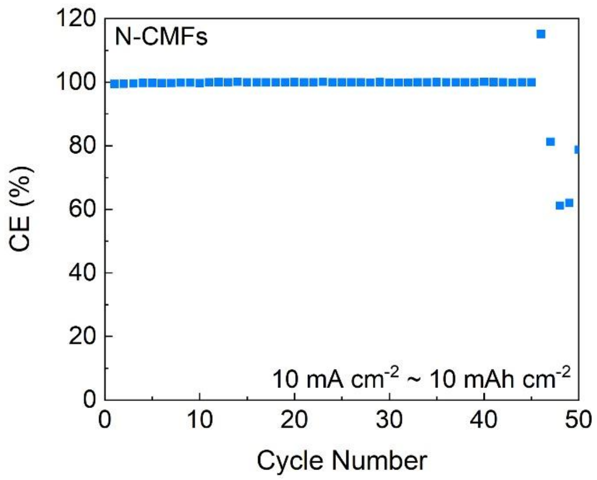

Fig. S31. CE analysis. CE plot of N-CMFs tested at 10 mA cm^{-2} and 10 mAh cm^{-2}.

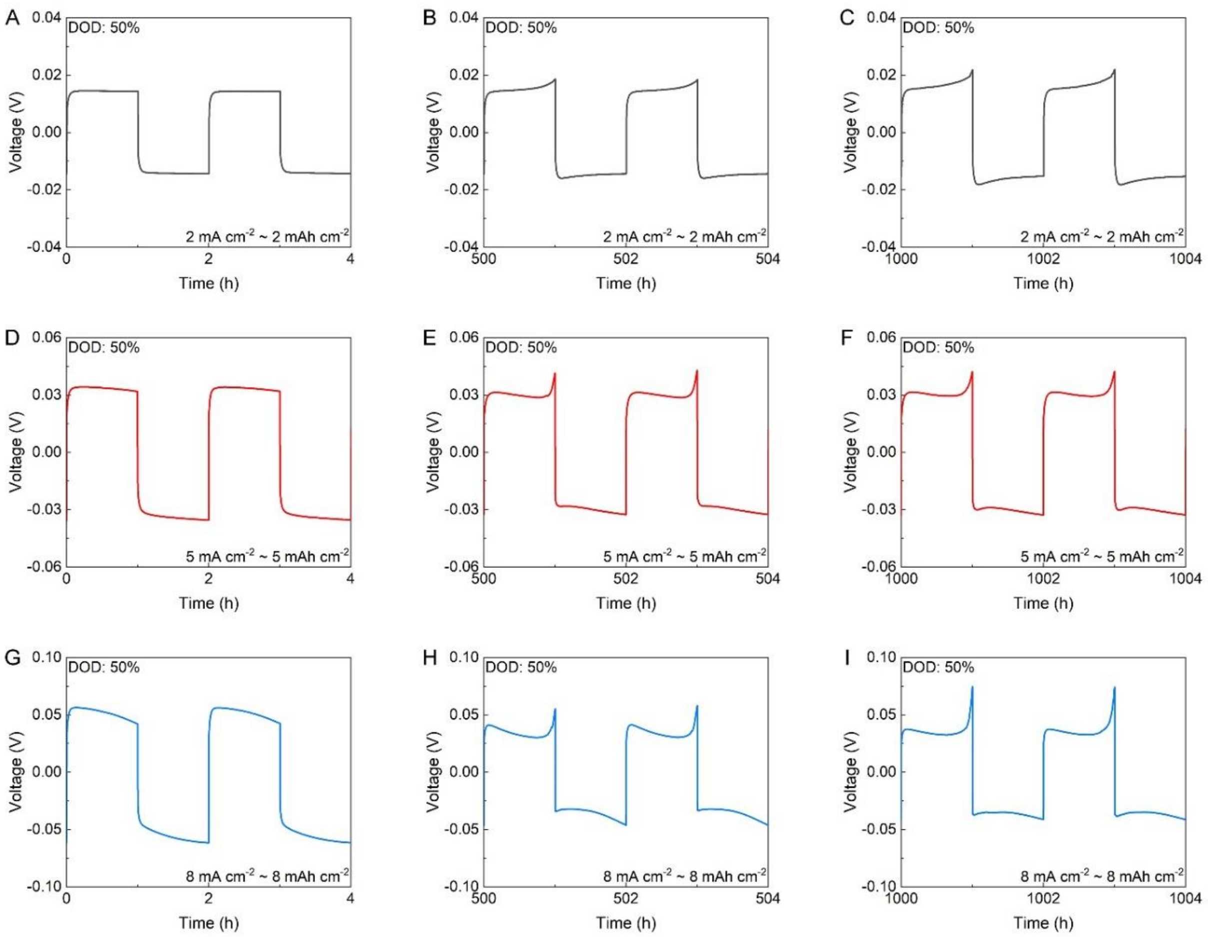

Fig. S32. Cycle analysis. Enlarged voltage profiles of CoP@N/P-CMFs-Na tested at various current densities and areal capacities. (**A**-**C**) 2 mA cm^{-2} and 2 mAh cm^{-2}, (**D**-**F**) 5 mA cm^{-2} and 5 mAh cm^{-2}, (**G**-**I**) 8 mA cm^{-2} and 8 mAh cm^{-2}.

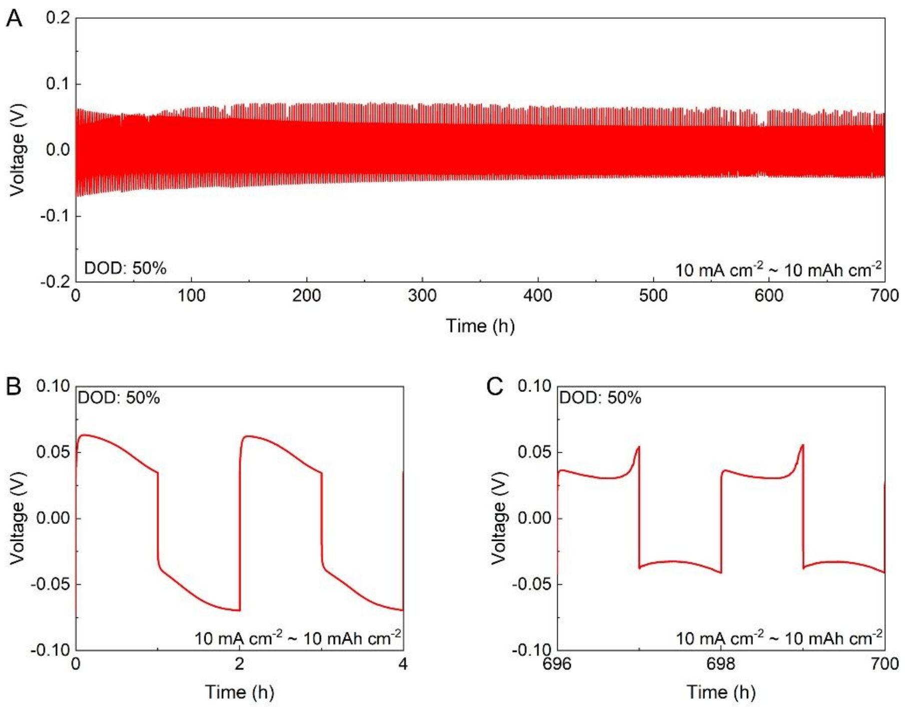

Fig. S33. Cycle analysis. (**A**) Cycling performance and (**B** and **C**) enlarged voltage profiles of CoP@N/P-CMFs-Na tested at 10 mA cm^{-2} and 10 mAh cm^{-2}.

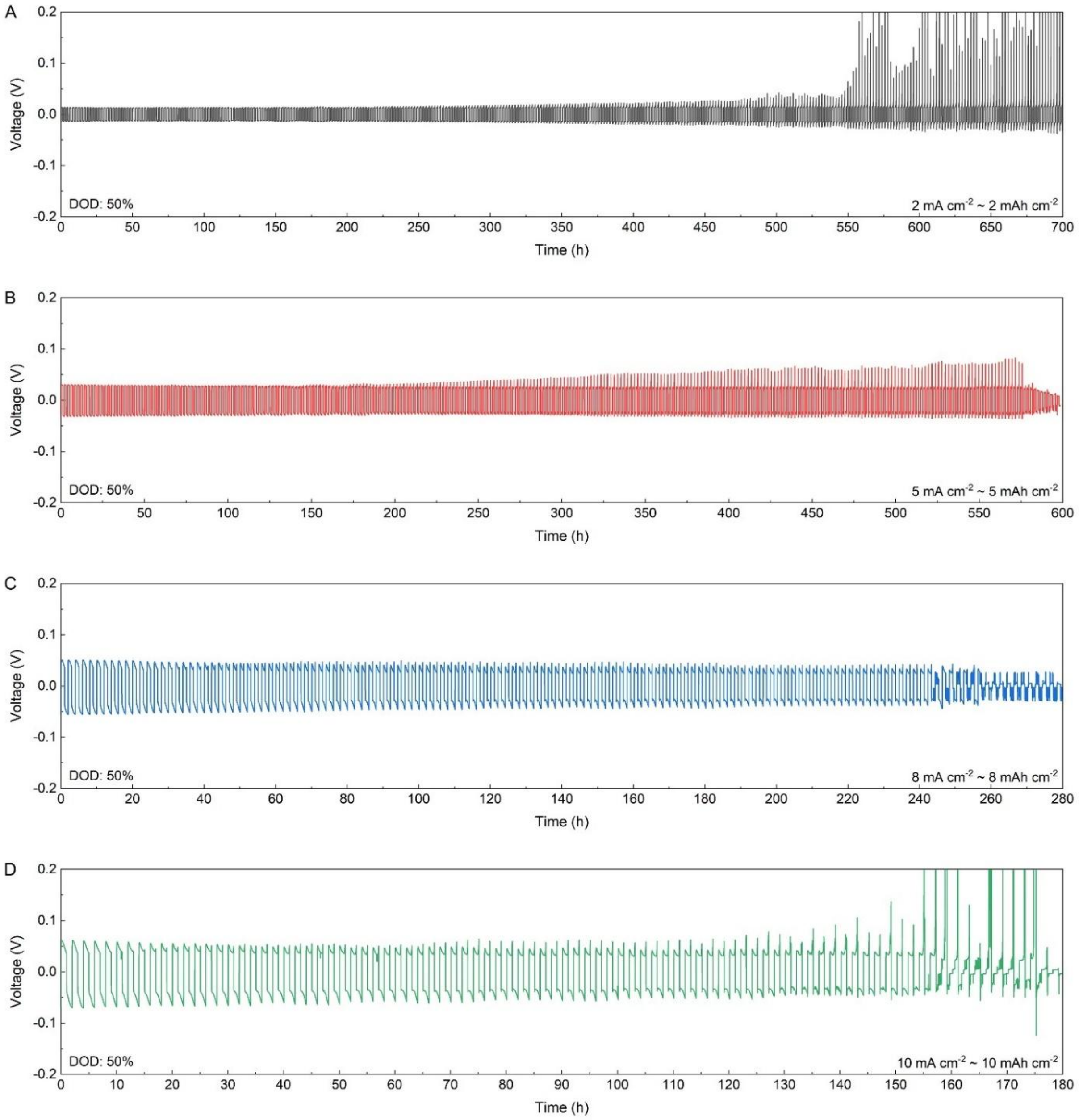

Fig. S34. Cycle analysis. Cycling performance of N-CMFs-Na tested at different current densities and areal capacities: (**A**) 2 mA cm^{-2} and 2 mAh cm^{-2}, (**B**) 5 mA cm^{-2} and 5 mAh cm^{-2}, (**C**) 8 mA cm^{-2} and 8 mAh cm^{-2}, (**D**) 10 mA cm^{-2} and 10 mAh cm^{-2}.

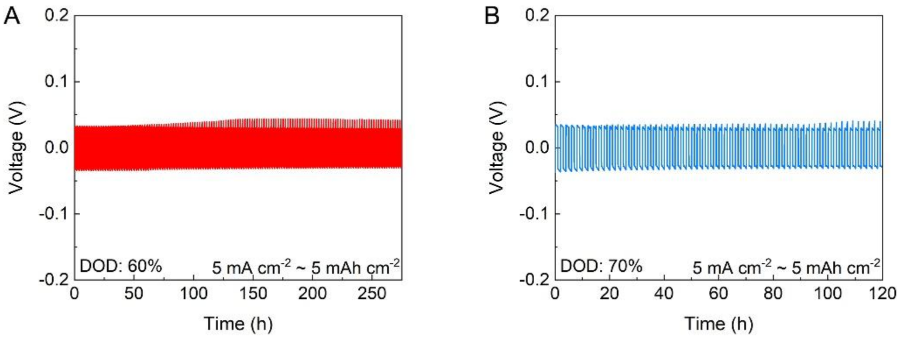

Fig. S35. Cycling analysis. Cycling performance of CoP@N/P-CMFs-Na tested at 5 mA cm^{-2} and 5 mAh cm^{-2} at high DOD values of (**A**) 60% and (**B**) 70%.

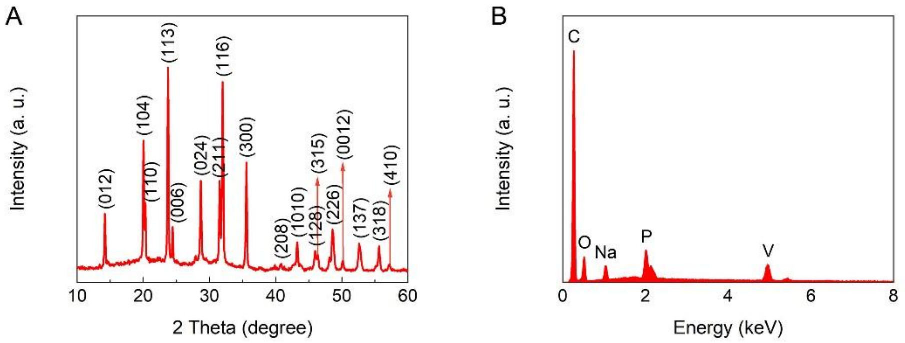

Fig. S36. XRD and EDX measurements. (**A**) XRD pattern and (**B**) EDX spectrum of NVP.

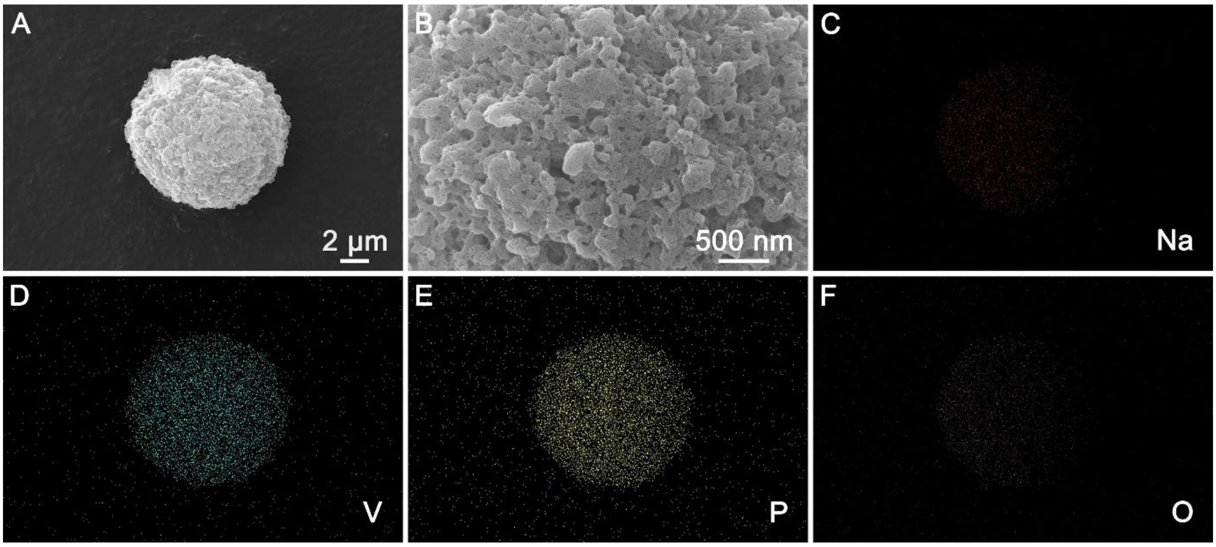

Fig. S37. FESEM and EDS measurements. (**A** and **B**) FESEM and (**C** to **F**) EDS mapping images of NVP.

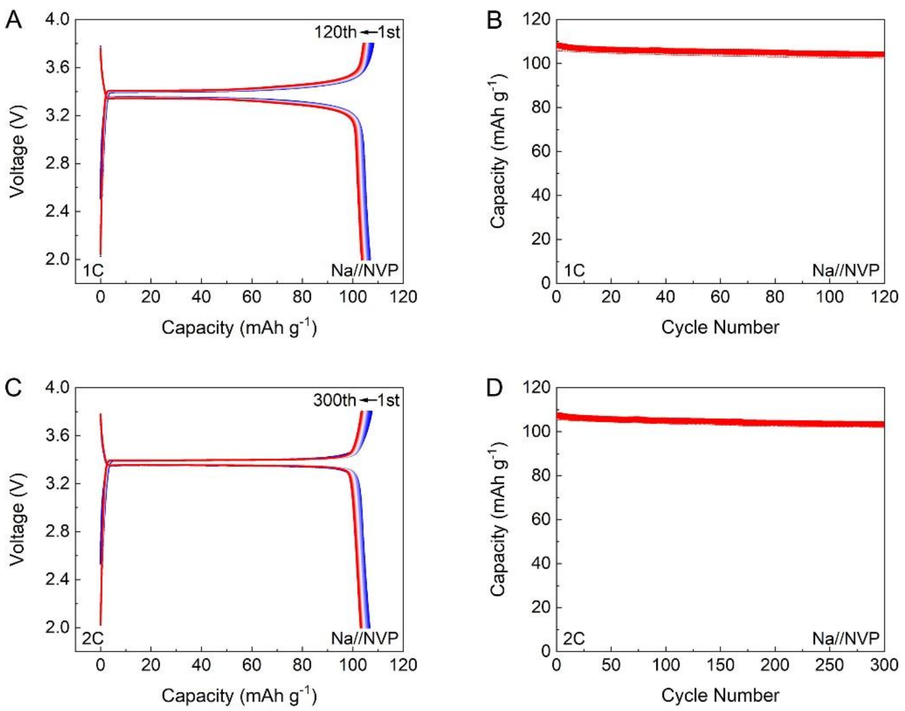

Fig. S38. Cycling tests. (**A** and **C**) Charge-discharge voltage profiles and (**B** and **D**) cycling performance of Na//NVP cells tested at (**A** and **B**) 1C and (**C** and **D**) 2C.

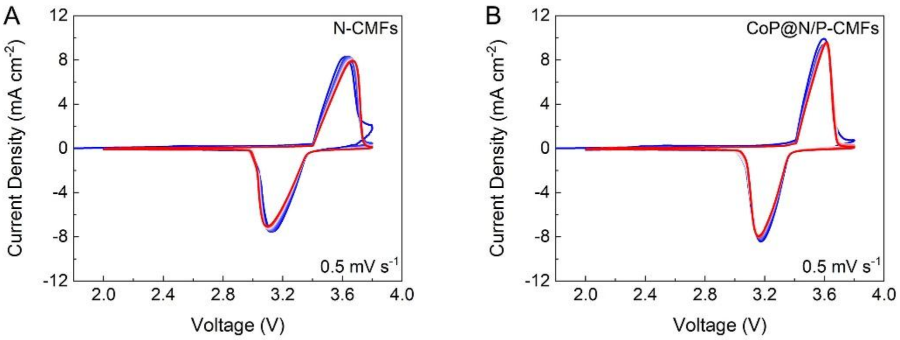

Fig. S39. CV analysis. CV curves of (**A**) N-CMFs//NVP and (**B**) CoP@N/P-CMFs//NVP cells tested at a scan rate of 0.5 mV s^{-1}.

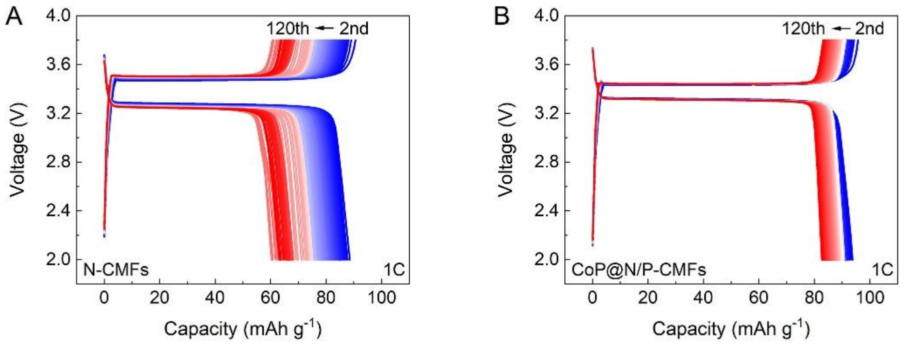

Fig. S40. Cycling tests. Charge-discharge voltage profiles from 2nd to 120th of (**A**) N-CMFs//NVP and (**B**) CoP@N/P-CMFs//NVP cells tested at 1C.

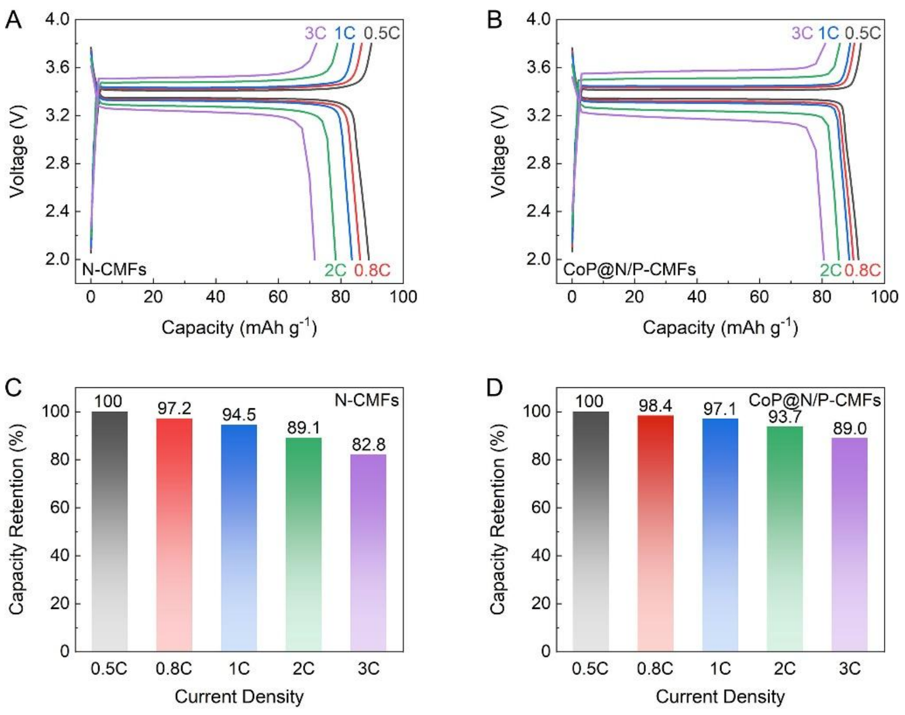

Fig. S41. Rate analysis. (**A** and **B**) Charge-discharge voltage profiles and (**C** and **D**) capacity retentions of (**A** and **C**) N-CMFs//NVP and (**B** and **D**) CoP@N/P-CMFs//NVP cells tested at different current densities.

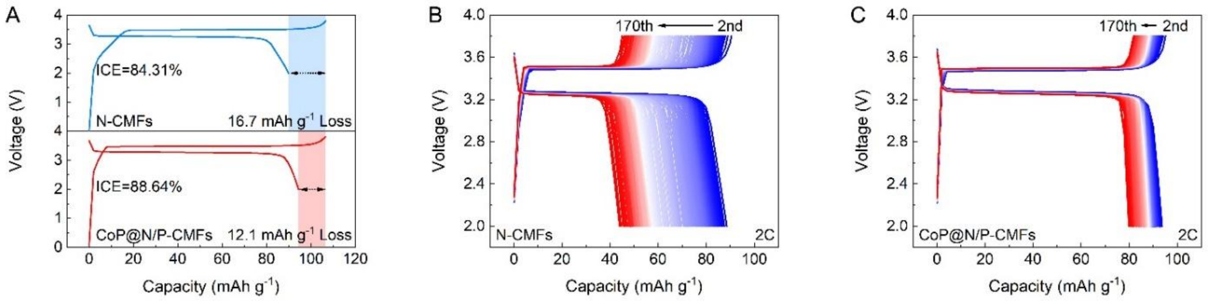

Fig. S42. Cycling analysis. (**A**) First charge-discharge voltage profiles, and (**B** and **C**) charge-discharge voltage profiles from 2nd to 170th of (**B**) N-CMFs//NVP and (**C**) CoP@N/P-CMFs//NVP cells tested at 2C.

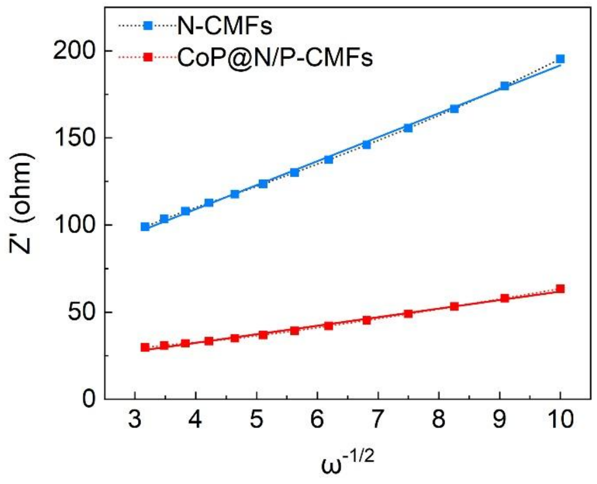

Fig. S43. EIS analysis. Relationship plots between *Z*' and $\omega^{-1/2}$ of N-CMFs//NVP and CoP@N/P-CMFs//NVP cells after cycling.

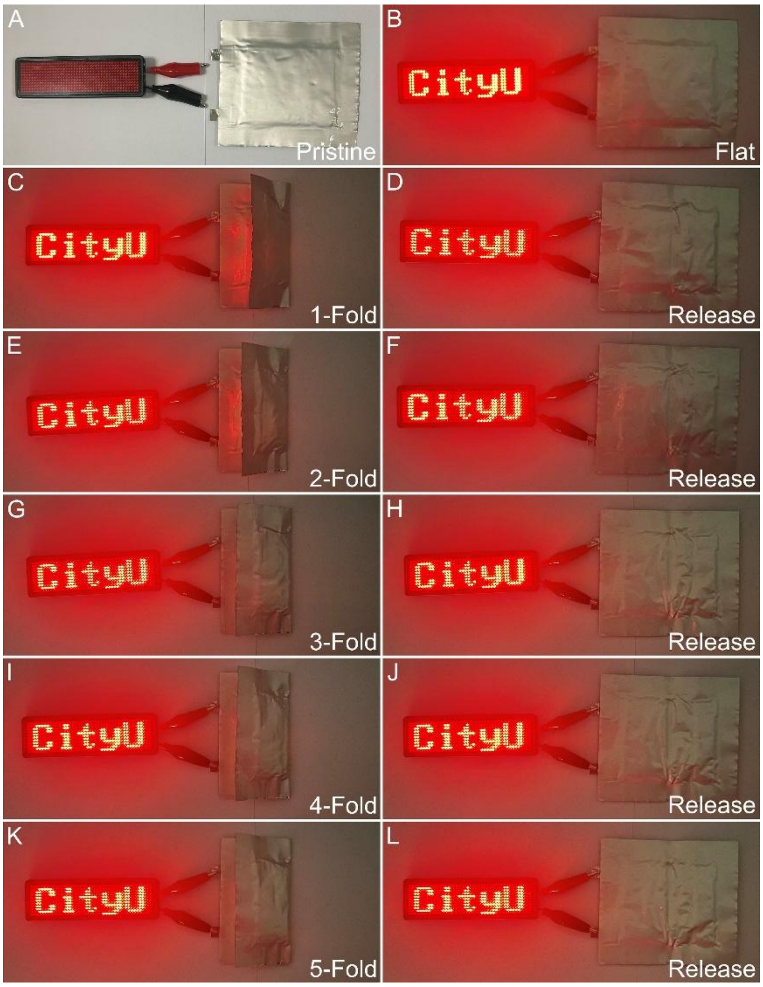

Fig. S44. Flexibility analysis. Demonstrations of practical application by lighting LEDs at different mechanical deformations: (**A**) pristine, (**B**) flat, (**C**) 1-fold, (**D**) release after 1-fold, (**E**) 2-fold, (**F**) release after 2-fold, (**G**) 3-fold, (**H**) release after 3-fold, (**I**) 4-fold, (**J**) release after 4-fold, (**K**) 5-fold, and (**L**) release after 5-fold.

3. Tables in the Supplementary Materials.

Table S1. Comparisons of Na plating/stripping performance of CoP@N/P-CMFs with different current densities and areal capacities.

Current Density (mA cm^{-2})	Areal Capacity (mAh cm^{-2})	Cycle Number	Time (h)	Accumulated Capacity (mAh cm^{-2})	CE (%)
1	1	480	960	480	99.58
2	1	900	900	900	99.86
5	1	900	360	900	99.84
10	1	5700	1140	5700	99.98
5	2	720	576	1440	99.92
5	5	330	660	1650	99.92
5	8	207	662.4	1600	99.96
5	10	500	2000	5000	99.96
10	10	330	660	3300	99.97

Table S2. Comparisons of Na plating/stripping performance of the CoP@N/P-CMFs host and other hosts reported in previous works.

Electrode	Electrolyte	Current Density (mA cm^{-2})	Areal Capacity (mAh cm^{-2})	Cycles	Accumulated Capacity (mAh cm^{-2})	CE (%)	Ref.
Al	Na-SSZE	1	1	1000	1000	99.84	(*17*)
Cu_3P@Cu	1M $NaPF_6$ in Diglyme	4	4	250	1000	99.12	(*24*)
FCTF	1M $NaPF_6$ in Diglyme	2	1	400	400	99.6	(*19*)
SnNCNFs	1M $NaPF_6$ in Diglyme	3	3	2000	6000	99.96	(*21*)
N-CSs/Cu	1M $NaPF_6$ in Diglyme	2	2	900	1800	99.99	(*50*)
Al-Cu@C	1M $NaPF_6$ in Diglyme	0.5	1	60	60	97.5	(*51*)
PC-CFe	1M $NaPF_6$ in Diglyme	10	10	500	5000	99.6	(*20*)
Cu@Au	1M $NaSO_3CF_3$ in Diglyme	2	2	300	600	99.8	(*52*)
Porous Al	1M $NaPF_6$ in Diglyme	1	0.5	1000	500	99.8	(*28*)
Carbon/Al	1M $NaPF_6$ in Diglyme	0.5	0.25	1000	250	99.8	(*53*)
3D Zn@Al	1 M $NaPF_6$ + DME	2	2	500	1000	99.5	(*54*)
O-CCF	1M $NaPF_6$ in Diglyme	5	10	1000	10000	99.6	(*15*)
Cu	1M $NaPF_6$ in Diglyme + SiO_2	1	2	500	1000	-	(*55*)
Sb_2MoO_6	1M $NaPF_6$ in Diglyme	5	4	500	2000	95.2	(*56*)
OCF	0.01 M NaTFSI + 1M $NaSO_3CF_3$ in Diglyme	10	1	2500	2500	99.83	(*57*)
a-CNTs	1M $NaSO_3CF_3$ in Diglyme	3	1	1000	1000	99.8	(*58*)
MgF_2@RGO	1M $NaClO_4$ in EC/DEC + 5% FEC	0.5	0.5	325	162.5	~96	(*59*)
3D-NVP	1M $NaClO_4$ in PC + 5% FEC	1	1	190	190	98	(*60*)
2D Sn/C	1M $NaSO_3CF_3$ in Diglyme	2	2	970	1940	99.85	(*61*)
CT-Sn(II)@Ti_3C_2	1M $NaPF_6$ in Diglyme	10	3	100	300	98.5	(*62*)
Cu@Sn	0.01M NaTFSI + 1M NaOTf in Diglyme	2	1	2000	2000	99.9	(*36*)
Cu@Sb	0.01M NaTFSI + 1M NaOTf in Diglyme	2	1	1600	1600	99.9	(*36*)
At-Sn@HCN	1M $NaPF_6$ in Diglyme	2	1	1000	1000	99.93	(*63*)
NiSb-CC	1M $NaClO_4$ in EC/DEC + 5% FEC	-	3.5	150	525	99.74	(*64*)
NSCNT	1M $NaSO_3CF_3$ in Diglyme	1	1	400	400	99.82	(*65*)

Electrode	Electrolyte	Current Density (mA cm^{-2})	Areal Capacity (mAh cm^{-2})	Cycles	Accumulated Capacity (mAh cm^{-2})	CE (%)	Ref.
L700	1M $NaSO_3CF_3$ in Diglyme	1	2	300	600	99.6	(*66*)
CoP@N/P-CMFs	1M $NaPF_6$ in Diglyme	10	1	5700	5700	99.98	This Work
		10	10	330	3300	99.97	

Table S3. Comparisons of cycling performance of CoP@N/P-CMFs-Na anodes with different current densities and areal capacities.

Current Density (mA cm^{-2})	Areal Capacity (mAh cm^{-2})	Depth-of-Discharge (%)	Cycle Number	Time (h)	Accumulated Capacity (mAh cm^{-2})
2	2	50	750	1500	1500
5	5	50	750	1500	3750
8	8	50	750	1500	6000
10	10	50	350	700	3500

Table S4. Comparisons of cycling performance of CoP@N/P-CMFs-Na and other composite Na anodes reported in previous works.

Electrode	Electrolyte	Current Density (mA cm^{-2})	Areal Capacity (mAh cm^{-2})	Depth-of-Discharge (%)	Accumulated Capacity (mAh cm^{-2})	Life (h)	Ref.
Cu_3P@Cu-Na	1M $NaPF_6$ in Diglyme	2	2	-	2000	2000	(*24*)
FCTF-Na	1M $NaPF_6$ in Diglyme	2	2	100	140	140	(*19*)
HCOONa	1M $NaPF_6$ in Diglyme	2	1	-	2200	2200	(*25*)
SnNCNFs-Na	1M $NaPF_6$ in Diglyme	10	10	86	3500	700	(*21*)
N-CSs-Na	1M $NaPF_6$ in Diglyme	2	2	-	1500	1500	(*50*)
NST-Na	1M $NaPF_6$ in Diglyme	2	10	60	1500	1500	(*67*)
PC-CFe-Na	1M $NaPF_6$ in Diglyme	5	5	-	4125	1650	(*20*)
Porous Al-Na	1M $NaPF_6$ in Diglyme	0.5	0.5	25	250	1000	(*28*)
3D Zn@Al-Na	1 M $NaPF_6$ + DME	2	1	50	1500	1500	(*54*)
O-CCF-Na	1M $NaPF_6$ in Diglyme	50	1	16.67	10000	400	(*15*)
F-A-Al-Na	Na[FSA] in [C2C1im][FSA]	0.5	0.5	25	150	600	(*18*)
Sb_2MoO_6-Na	1M $NaPF_6$ in Diglyme	10	8	-	960	192	(*56*)
OCF-Na	0.01 M NaTFSI + 1.0 M CF_3NaO_3S in Diglyme	5	5	-	5000	2000	(*57*)
a-CNTs-Na	1M $NaSO_3CF_3$ in Diglyme	5	8	-	750	300	(*58*)
MgF_2@RGO-Na	1M $NaClO_4$ in EC/DEC + 5% FEC	0.5	0.5	10	400	1600	(*59*)
3D-NVP-Na	1M $NaClO_4$ in PC + 5% FEC	1	2	20	200	400	(*60*)
2D Sn/C-Na	1M $NaSO_3CF_3$ in Diglyme	3	3	50	150	100	(*61*)
CT-Sn(II)@Ti_3C_2-Na	1M $NaPF_6$ in Diglyme	5	3	60	600	240	(*62*)
At-Sn@HCN-Na	1M $NaPF_6$ in Diglyme	4	8	80	10000	5000	(*63*)
NiSb-CC-Na	1M $NaClO_4$ in EC/DEC + 5% FEC	1	10	47	500	1000	(*64*)
NSCNT-Na	1M $NaSO_3CF_3$ in Diglyme	1	1	-	250	500	(*65*)
L700-Na	1M $NaSO_3CF_3$ in Diglyme	1	0.5	-	500	1000	(*66*)

Electrode	Electrolyte	Current Density (mA cm^{-2})	Areal Capacity (mAh cm^{-2})	Depth-of-Discharge (%)	Accumulated Capacity (mAh cm^{-2})	Life (h)	Ref.
CoP@N/P-CMFs-Na	1M $NaPF_6$ in Diglyme	8	8	50	4000	1000	This Work
		10	10	50	3500	700	

Table S5. Comparisons of electrochemical performance of recently reported anode-free Na batteries.

Cathodes	Mass Loading (mg cm^{-2})	Anodic Current Collector	Cut-off Voltage (V)	Current Density (mA g^{-1})	Cycles	Capacity (mAh g^{-1})	Capacity Retention (%)	Ref.
NVPOF	7.1	C@Al	2.0-4.25	0.4 mA cm^{-2}	370	97.9	89.2	(*17*)
NVP	13.6	Cu_3P@Cu	2.5-3.5	60	75	76.1	-	(*24*)
NVP	7.4	FCTF	2.5-3.8	240	400	-	56	(*19*)
NVP	10	SF-Cu	2.8-3.7	58.5	400	86.3	88	(*25*)
NVP	10	SnNCNF	2.6-3.8	234	80	89.3	89	(*21*)
NNMO	4	N-CPs	2.5-3.8	50	200	-	86.3	(*50*)
NVP	2.5	NST	2.2-3.8	118	100	73.15	77	(*67*)
NNCFM	8.52	C@Al	2.0-4.0	30	250	63.9	-	(*30*)
NCNFM	15.12	GC@Al	2.0-3.8	63.5	260	-	84	(*14*)
NVP/C	3.5-4.0	Al-Cu@C	1.5-3.8	120	50	-	-	(*51*)
NVP	10	PC-CFe	2.6-3.8	1 mA cm^{-2}	100	~103	97	(*20*)
Na-FeS_2	2	Cu@Au	0.8-3.0	-	50	140	-	(*52*)
NVP	12	CB/Al	3.0-3.7	-	100	-	82.5	(*29*)
Na-TiS_2	-	Porous Al	1.3-2.6	0.1 mA cm^{-2}	200	~160	~64	(*28*)
$Na_{1.5}FeS_2$	5	Carbon/Al	0.8-3.0	0.125 mA cm^{-2}	40	335	-	(*53*)
NVP	11	O-CCF	2.5-4.0	1 mA cm^{-2}	100	103.7	96	(*15*)
NVP	~4	Cu	2.6-3.8	0.5 mA cm^{-2}	100	71.88	75	(*55*)
NVP	11.3	F-A-Al	2.4-3.6	35.1	50	-	46.1	(*18*)
NVP	18	CoP@N/P-CMFs	2.0-3.8	240	170	79.88	84.63	This Work
